# Variable task difficulty with augmented sensory feedback for motor training in virtual reality

**DOI:** 10.3389/fbioe.2026.1884697

**Published:** 2026-10-05

**Authors:** Yu Shi, Sophie Dewil, Zachary Marvin, Noam Y. Harel, Raviraj Nataraj

**Affiliations:** 1 Department of Biomedical Engineering, Stevens Institute of Technology, Hoboken, NJ, United States; 2 Movement Control Rehabilitation (MOCORE) Laboratory, Altorfer Complex, Stevens Institute of Technology, Hoboken, NJ, United States; 3 Spinal Cord Damage Research Center, James J. Peters VA Medical Center, Bronx, NY, United States; 4 Departments of Neurology and Rehabilitation & Human Performance, Icahn School of Medicine, New York, NY, United States

**Keywords:** augmented sensory feedback, movement rehabilitation, perceptual, physiological response, task difficulty, virtual reality

## Abstract

**Introduction:**

This study examined how motor task difficulty and augmented sensory feedback (ASF) modality influence task performance, physiological responses, and perceptual experience during virtual reality (VR)-based upper-limb motor training. Building on related brace- and VR-based ASF platforms previously examined in neurotypical and spinal cord injury cohorts, the long-term goal is to support neurorehabilitation-oriented adaptive VR training; however, the present study used neurotypical participants as an initial validation step.

**Methods:**

Fourteen right-handed neurotypical participants used a supportive upper-limb brace and surface electromyography (EMG) for myoelectric control of a virtual robotic arm in a two-dimensional tracking task. A 2 × 2 within-subject design crossed ASF modality (visual feedback versus visual + haptic feedback) with task difficulty (straight-line versus sinusoidal trajectory). Outcomes included normalized path length, task completion time, movement jerk, alpha- and beta-band electroencephalogram (EEG) power over motor cortex, heart rate, EMG amplitude, EMG-displacement-based virtual work, and perceptual ratings of agency, motivation, and utility. Short-term pre-to-post percentage changes were computed for each measure.

**Results:**

VHF straight-line training produced the largest reductions in normalized path length (-4.69%) and completion time (-6.12%), whereas VHF sinusoid training produced the largest reductions in movement jerk (-5.67%) and virtual work (-13.33%). ASF modality affected path length, completion time, jerk, EMG amplitude, and virtual work, whereas task difficulty modulated beta-band EEG power and the physiological index. Changes in path length were positively associated with changes in completion time and EMG amplitude.

**Discussion:**

These findings suggest that ASF modality and task difficulty can differentially shape short-term behavioral, physiological, and perceptual responses in healthy participants. The exploratory distribution of participant-specific best-case conditions motivates future studies using independent trial subsets to evaluate whether behavioral and physiological measures can support individualized VR training.

## 1 Introduction

Neurological injuries or disorders, such as spinal cord injury, frequently result in severe motor impairments that compromise individuals’ independence in activities of daily living (ADL) and overall quality of life (Colantonio et al., 2004; Lili et al., 2024). Traditional physical and occupational therapy, widely employed for motor rehabilitation, often focuses on upper-extremity function due to its critical role in daily activities such as feeding and dressing (Behrman et al., 2017; Hoffman and Field-Fote, 2010). However, therapy relies heavily upon repetitive and intensive training paradigms (Inanici et al., 2021), which can diminish long-term patient adherence due to declining motivation and fatigue, thus limiting therapeutic dosage. Innovative rehabilitation approaches are therefore needed not only to sustain patient engagement, but also to enable more quantitative, controllable, and individualized evaluation of motor training.

Technology-based solutions are increasingly used to address limitations in traditional therapy. Virtual reality (VR) has shown substantial potential for motor rehabilitation due to its programmable and immersive characteristics. Prior studies have demonstrated that VR-based rehabilitation can markedly increase patient interest and engagement through interactive virtual scenarios and gamified design elements (Sveistrup, 2004; Prasad et al., 2018). However, prior meta-analyses suggest that when the training dosage is matched between VR and traditional physical therapy, the added therapeutic benefits offered by VR have been negligible (Wälchli et al., 2016; Laver et al., 2017). This suggests that the programmable and personalized capabilities of VR technology have not yet been fully realized. In addition to improving engagement, VR may also reduce experimental bias associated with boredom and motivation loss during repetitive motor-training sessions (Pasquini et al., 2026). Within this clinical motivation, the present study should be viewed as an initial validation of training-parameter effects in neurotypical participants, rather than a direct test of rehabilitation efficacy.

A promising avenue for customizing VR-based motor training is augmented sensory feedback (ASF), which provides additional sensory guidance beyond what is naturally available to the user (Simone et al., 2007). Recent work using an intelligent glove integrated with VR further demonstrated the feasibility of augmented-feedback-based functional grasp training in persons with cervical-level spinal cord injury, supporting the clinical relevance of programmable VR systems for upper-extremity rehabilitation (Liu et al., 2026). ASF can be implemented through activation of individual sensory modalities (e.g., visual or haptic) alone or in combination (multimodal feedback). In VR-based rehabilitation, visual augmented feedback is especially beneficial, as it provides either real-time or post-task cues for movement correction, spatial orientation, and achievement of target movement features (Simone et al., 2007; Liu et al., 2021; Sanford et al., 2021). Multimodal feedback, defined as the concurrent activation of multiple sensory modalities to cue the same information about performance, has been shown to accelerate motor learning (Sigrist et al., 2013). Such feedback expedites the crossing of neural activation thresholds associated with motor learning and better reflects the multisensory character of daily physical interaction (Seitz and Dinse, 2007). Beyond sensory modality alone, recent proof-of-concept work also suggests that pairing ASF with memory-anchored cognitive cues can improve hand-gesture acquisition, accuracy, execution speed, and kinematic stability, indicating that cognitive scaffolding may be another design dimension for VR-based motor training (Marvin et al., 2026). However, the benefits of ASF are contingent on delivering an appropriate “dose” and selecting an effective delivery strategy, both of which shape individual responsiveness and ultimate functional gains. For example, excessive or overly complex feedback may induce cognitive overload and thereby impair learning by overtaxing attentional and working-memory resources (Sweller, 2011). Although rehabilitation must promote sufficient physical effort, consistent with the principle of “no pain, no gain” (Boyne et al., 2023), it is equally important to prevent premature physical and mental fatigue, which may increase injury risk or constrain the capacity to regain better function (Keller et al., 2024). Accordingly, training should achieve an optimal balance such that individuals remain adequately engaged without being overwhelmed by training elements including task difficulty (Woodbury et al., 2016; Guadagnoli and Lee, 2004) and, in this context, feedback guidance. However, it remains unclear how different ASF modalities influence not only motor-task performance but also the broader set of psychophysiological responses that index underlying bodily (physical) and cognitive (mental) states, which are fundamental for effective motor behavior. These considerations point to a central design question for VR-based motor training: how sensory guidance and task challenge together shape not only task performance, but also the physiological and perceptual responses that may support individualized training.

A variety of psychophysiological measures, reflecting domains such as neural engagement, affective arousal, physical effort, and subjective experience, have been linked to better performance (Afrash et al., 2023; Decety and Jackson, 2004). Noninvasive skin-surface recordings capturing brain, cardiac, electrodermal, and muscular activity can serve as robust indicators of physiological processing during neuromotor rehabilitative training, especially when training is delivered through computerized interfaces. Within virtual environments, electroencephalography (EEG) can capture participant states associated with mental workload and attention (Frey et al., 2016) as well as emotional arousal (Hofmann et al., 2021). Heart rate (HR) provides a strong correlate of sympathetic nervous system activation and is also commonly used as a proxy for cognitive and emotional load (Nasri, 2025). Finally, electromyography (EMG) offers a direct index of task-related muscular effort during motor performance (Reaz et al., 2006).

Beyond physiological measures, the sense of agency is a fundamental component of human motor experience (Moore, 2016), reflecting the subjective feeling of voluntary control and commonly indexed using paradigms such as intentional binding in simple actions (e.g., key presses) (Haggard et al., 2002). Agency is known to be altered in individuals with neurological or cognitive impairments (Kranick et al., 2013; Moore et al., 2011) and has been extensively studied in computerized and virtual-reality environments, where control and embodiment can be systematically manipulated (Ziadeh et al., 2021; Girondini et al., 2025). Prior work further indicates that agency (intentional binding) is positively associated with motor performance during interactive tasks (Nataraj et al., 2020a; Nataraj et al., 2020b; Nataraj and Sanford, 2021), and that agency can increase during VR-based motor rehabilitation (Aoyagi et al., 2019). Beyond agency, perceptual factors such as motivation (Li et al., 2024) and perceived utility (Grevet et al., 2023) are also critical for evaluating the clinical effectiveness and acceptability of emerging rehabilitation approaches.

In previous studies using the same supportive upper-limb brace adopted in this study (Sanford et al., 2022; Shi et al., 2024; Nataraj et al., 2025; 2026), multiple forms of ASF were examined. The platform relies on myoelectric control, in which surface EMG signals are decoded to command movements of virtual avatars while the participant’s arm is supported in place against gravity. Myoelectric control has become an important component of rehabilitation-oriented human–machine interfaces because it enables residual voluntary muscle activity to be translated into user-intended control commands for assistive devices, rehabilitation robots, and virtual avatars (Liu J. and Zhou, 2013). In robot-aided rehabilitation, EMG-driven hand exoskeletons and combined neuromuscular electrical stimulation–robot systems have been developed to detect voluntary motor intent and provide task-specific assistance during hand opening, closing, and tracking exercises (Ho et al., 2011). More broadly, adaptive and assist-as-needed rehabilitation robots can provide intensive and repeatable motor practice while modifying the level of assistance according to participant performance or motor capability (Masia et al., 2009; Squeri et al., 2014; Zhang et al., 2020). In parallel, EMG-driven VR interfaces translate muscle activity into control of virtual limbs or end effectors, allowing task parameters and augmented feedback to be manipulated without physically actuating the participant’s limb (Sanford et al., 2022; Shi et al., 2024; Nataraj et al., 2025). Such decoding commonly relies on machine-learning methods, which learn mappings between physiological signal features and intended motor outputs. In the present framework, SVM-based regression provided a practical approach for converting multichannel EMG features into continuous two-dimensional control commands. Thus, the present system shares the intent-driven and active-participation principles of EMG-driven robotic rehabilitation, but it uses a virtual robotic arm rather than a physically actuated rehabilitation robot that provides mechanical assistance or resistance.

Compared with the baseline condition (without receiving ASF), participants broadly exhibited improvements in motor performance under ASF-enhanced conditions for both neurotypical participants and those with cervical-level incomplete spinal cord injury. Notably, visual feedback (VF) produced the largest improvements in motor performance alongside reduced EMG activity, whereas physiological arousal increased, as reflected by enhanced electrodermal activity. In contrast, multimodal ASF (visual + haptic feedback, VHF) elicited stronger cortical engagement, including significant post-training increases in EEG alpha- and beta-band power, as well as higher motivation and utility ratings. Related work from the authors’ group has also examined how VR-based motor training can integrate proactive and reactive task constraints with augmented auditory feedback to shape tracing accuracy, movement variability, and physiological arousal (Dewil et al., 2026). Collectively, these findings suggest that ASF modality differentially shapes motor, physiological, and perceptual responses, states that are closely tied to effective motor behavior. However, these studies did not examine how ASF interacts with task difficulty, nor did they extend evaluation to movement-quality or effort-related descriptors.

According to motor learning theory, optimal skill acquisition occurs when the difficulty of the task dynamically matches the learner’s evolving capability (Woodbury et al., 2016). Accordingly, task difficulty analysis is clinically relevant because it can help quantify how individuals adapt to changing motor demands and may inform how challenge levels are adjusted during training. Prior visuomotor-adaptation studies have used perturbation-based paradigms to assess adaptation in healthy participants, older adults, and individuals with movement disorders (Xing and Jeffrey, 2021; Osaba et al., 2020). Excessive difficulty can induce cognitive overload, typically reflected by elevated EEG activity in the alpha band (Li et al., 2025), whereas insufficient challenge fails to engage adaptive neural resources sufficiently (Nesbitt, 2003). In VR-based therapy, task difficulty can be defined and modified across kinematic, temporal, sensory, and cognitive dimensions to approximate an individual’s optimal challenge point. In trajectory-control motor tasks, difficulty can be systematically manipulated by altering target-path geometry, including trajectory curvature, path length, and required tracking speed. Such manipulations are particularly feasible in VR because digital environments allow precise control over movement demands. In a prior trajectory-tracking study, movement precision was used as an adaptive criterion for adjusting trajectory complexity (Squeri et al., 2014; Masia et al., 2009; Falzarano et al., 2019). Furthermore, several studies have employed error augmentation along predefined trajectories to amplify deviations, which, if applied at an appropriate dosage, can enhance motor learning in certain cohorts (Abdollahi et al., 2011; Liu et al., 2018). Overall, trajectory-based difficulty modulation provides an effective means to balance spatial precision and movement vigor while maintaining user autonomy, making it highly suitable for upper-limb VR trajectory-tracking rehabilitation (Zhang et al., 2020).

Coordinating task difficulty with sensory guidance offers a promising strategy to optimize VR-based rehabilitation training. Changes in task difficulty (e.g., from straight-line to sinusoidal trajectories) require corresponding adjustments in ASF to prevent performance plateaus that may signal the onset of physical or cognitive limits. Physiological measures are indicators of physical arousal and fatigue that can be scaled according to task difficulty and dynamically balanced against performance effort (Wulf and Shea, 2002; Charles and Nixon, 2019; Veltman and Gaillard, 1998). Proper alignment of task difficulty and sensory feedback can therefore target cognitive and physiological states that potentially maximize motor performance, and related gains in function.

Whereas prior work has examined ASF and task difficulty independently (Sanford et al., 2022; Wulf and Shea, 2002), their combined effects in VR rehabilitation remain underexplored. The associated physiological and cognitive consequences remain unclear, motivating further investigation to better incorporate these individualized psychophysiological responses into rehabilitative training to improve outcomes. To address this gap, the present study investigated how ASF modality and trajectory-based task difficulty influence short-term motor performance, movement quality, physiological responses, and perceptual experience within a VR-based upper-extremity motor-training platform. Based on prior ASF-enhanced VR training studies, it was hypothesized that multimodal ASF would produce more favorable behavioral and task-cost outcomes than visual-only ASF, and that ASF modality and task difficulty would differentially shape behavioral, physiological, and perceptual responses. More broadly, this study examined whether training outcomes could be better interpreted through the combined consideration of sensory guidance and task challenge. Section 2 describes the experimental framework, protocol, and outcome measures; Section 3 presents the effects of ASF modality and task difficulty across behavioral, physiological, and perceptual domains; and Section 4 discusses the implications of these findings for adaptive VR-based motor training.

## 2 Methods

### 2.1 Experimental framework and overview (design, setup)

The general experimental framework is illustrated in Figure 1. Figure 1A presents the experimental setup and the integrated sensing and feedback components. Figure 1B summarizes the pre-training, training, and post-training protocol, including the 2 × 2 manipulation of ASF modality and trajectory difficulty. Figure 1C provides a high-level overview of the myoelectric-control and data framework, showing real-time EMG decoding for VR task control together with parallel signal acquisition, offline synchronization, and multidimensional outcome assessment. Neurotypical participants placed their dominant-side arm into a custom, position-adjustable support brace while interacting with a VR-based upper-extremity reaching task. The brace supported the arm against gravity and allowed participants to generate semi-isometric muscle activity for myoelectric control. The setup integrated VR task display, surface EMG acquisition, EEG recording, heart-rate monitoring derived from photoplethysmography (PPG) at the left earlobe, marker-based motion tracking, and vibrotactile stimulation for haptic feedback. Participant-specific surface EMG signals were decoded in real time using support vector machine (SVM) regression to drive two-dimensional movement of a virtual robotic-arm end effector within the VR workspace, enabling participants to contact highlighted targets under different sensory-feedback and trajectory-difficulty conditions. The VR scene and viewpoint remained stationary throughout the task. Participants remained seated with the dominant arm supported, and only the virtual end effector moved within the task workspace in response to decoded EMG commands. The sinusoidal condition was implemented by altering the geometry of the prescribed end-effector path, rather than through artificial scene locomotion, visual-field oscillation, or imposed viewpoint translation. In contrast, visual-field oscillation paradigms perturb the global optical flow or visual reference frame and have been used to elicit visuomotor entrainment, balance responses, and adaptation to visual perturbations (Franz et al., 2017; Thompson and Franz, 2017; Martelli et al., 2019). By keeping the visual reference frame stable and varying only the prescribed end-effector path, the present design was intended both to more directly isolate upper-extremity trajectory-planning and myoelectric-control demands and to limit additional visual–vestibular conflict, whole-body balance responses, and perceptual demands that could influence downstream behavioral and physiological outcomes. The stable-scene design was also intended to reduce the potential for dizziness or cybersickness during head-mounted display use. Because no formal cybersickness questionnaire was administered and display latency was not instrumentally measured, cybersickness- and latency-related effects were not evaluated in the present study.

**FIGURE 1:**
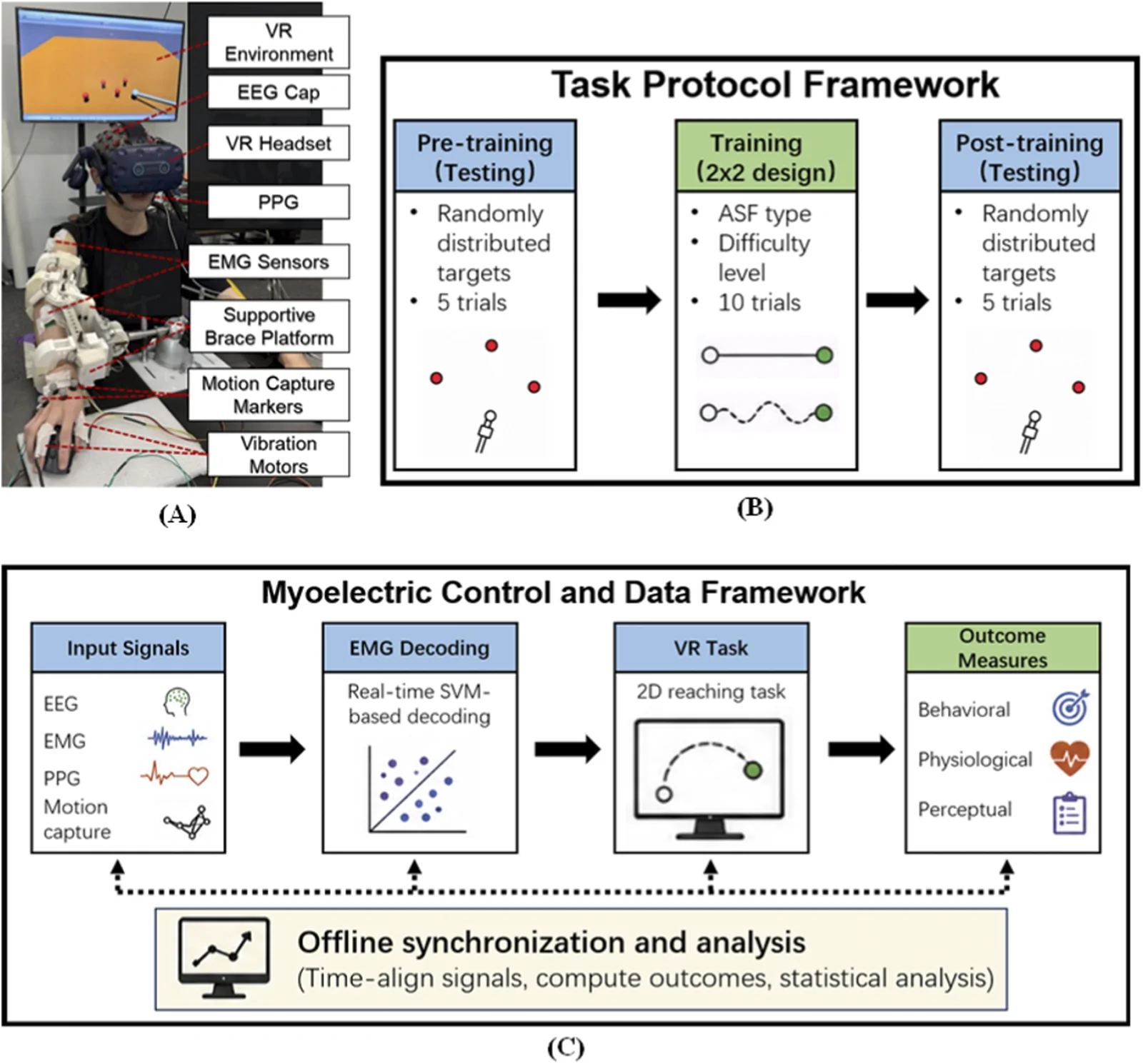
Experimental framework of the VR-based motor training system. **(A)** Experimental setup showing the seated participant interacting with the VR task while wearing the EEG cap, VR headset, PPG sensor, and surface EMG sensors, with the dominant arm supported by the custom brace platform and instrumented with motion-capture markers and vibration motors. **(B)** Task protocol framework consisting of five pre-training testing trials without ASF, ten training trials under one of four ASF–difficulty combinations, and five post-training testing trials without ASF. **(C)** Simplified myoelectric-control and data framework. Surface EMG was decoded in real time to control the virtual end effector, whereas physiological and kinematic signals were recorded during task performance. Relevant physiological and VR-derived signals were subsequently synchronized offline for computation of behavioral and physiological outcomes, which were evaluated together with perceptual measures.

Within this framework, the study investigated upper-extremity motor performance and psychophysiological responses under combined manipulations of ASF modality and task difficulty. A 2 × 2 within-subject design crossed ASF modality, visual feedback (VF) and visual + haptic feedback (VHF), with task difficulty, straight-line and sinusoidal tracking. Participants were instructed to reach all targets as efficiently as possible. Normalized movement path length served as the primary behavioral outcome, and task completion time served as the secondary behavioral outcome. The performance and neurophysiological effects of the different ASF–difficulty training combinations were quantified as mean pre-to-post changes by comparing five testing trials completed before training with five testing trials completed after training. During training, each block comprised 10 trials delivered under a specific ASF–difficulty combination designed to increase engagement and provide additional guidance during practice.

ASF was implemented to deliver real-time corrective information during trajectory tracking. Visual ASF was operationalized via a guidance sphere whose opacity scaled with deviation magnitude, whereas haptic ASF was conveyed through vibrotactile actuators on the hand that encoded both the direction and magnitude of trajectory error. Full details of each ASF condition are provided in Section 2.8. Task difficulty was operationalized by manipulating the geometry of the prescribed tracking trajectory, as trajectory complexity is an established means of modulating visual–motor task demand. The straight-line trajectory was defined as the easier level because it represented the shortest path between the start position and target and maintained a constant desired movement direction throughout the reach. In contrast, the sinusoidal trajectory was defined as the more difficult level because it was longer and imposed continuously varying curvature, requiring ongoing updates of movement direction and dynamic coordination of the two control axes to remain on the prescribed path. This distinction is consistent with prior motor-control evidence that curved trajectories differ from straight-line trajectories in their planning and execution, and with visual–motor tracking studies showing that greater target-trajectory complexity increases task demand and alters movement smoothness and neuromuscular activation (Abend et al., 1982; Wong et al., 2016; Deng et al., 2021).

Each training block consisted of ten trials delivered under one of four ASF–difficulty combinations. No ASF was provided during the five pre-training and five post-training testing trials. This protocol was designed to quantify short-term pre-to-post training-related changes and associated neurophysiological responses across distinct ASF training modalities.

### 2.2 Participant recruitment

This study enrolled a cohort of 14 neurotypical participants (4 males, 10 females; mean age 20.92 ± 2.84). All participants were right hand dominant as determined by verbal self-report. No standardized handedness scale was administered. During screening interviews, all participants confirmed the absence of (1) clinically identified neurological disorders affecting cognitive processing or motor function; (2) history of surgical procedures involving upper extremities or cervical/thoracic spinal regions; (3) auditory deficits uncorrectable to functional thresholds; visual impairments exceeding corrected acuity norms; or (4) photosensitivity-related seizure predisposition.

### 2.3 Skin-surface sensors for physiological recordings

A wireless surface EMG acquisition system (Trigno Wireless EMG System, Delsys Inc., Natick, MA, USA) recorded myoelectric signals from 14 muscle sites on the dominant upper extremity and ipsilateral torso (Figure 1A). Sensor placement targeted musculotendinous junctions of brachioradialis, digital extensors, brachial flexor-extensor complexes (biceps and triceps brachii), scapulothoracic stabilizers (trapezius divisions, infraspinatus, serratus anterior), and shoulder girdle actuators (latissimus dorsi, pectoralis major, deltoid subdivisions)—anatomical regions critical for upper limb kinematics and rehabilitation applications (Sanford et al., 2022; Shi et al., 2024; Keenan, 1988; Reinold et al., 2007). Surface EMG sensor placement followed SENIAM recommendations where applicable (Hermens et al., 2000). Before sensor placement, the skin was prepared to improve signal quality. Sensors were positioned over the corresponding muscle bellies using anatomical landmarks and palpation during voluntary contractions to confirm activation of the target muscle groups. The same sensor-placement scheme was maintained across all experimental conditions for each participant. EMG signals were acquired at 1925.925 Hz sampling frequency.

Brain activity was captured using a 64-electrode EEG scalp-recording cap (g.GAMMACAP, g.tec medical engineering GmbH, Austria) with 256 Hz sampling rate. The electrodes, spaced approximately 1 cm apart, had placements consistent with a modified (10-10) version of the extended international 10–20 system to accommodate 64 channels. These channels covered the primary motor cortex, premotor cortex, and posterior parietal cortex, regions associated with agency (Moore et al., 2010; Shu et al., 2018). Data were acquired using BCI2000 v3.6 beta with acquisition-stage filtering enabled, including a 0.1–60 Hz band-pass filter and a 58–62 Hz notch filter to reduce slow drift, high-frequency noise, and power-line contamination. EEG was recorded with FPz as the reference and a right earlobe electrode serving as ground. EEG preprocessing was limited to sensor-level artifact screening and epoch rejection before band-power analysis. Prior to spectral analysis, trial-level EEG segments were defined using a marker sequence generated exclusively by the VR task. The marker value increased by one whenever the virtual end effector contacted the currently active target. The same target-contact event changed the target color and activated the next target in the VR interface, as described in Section 2.7. Following offline synchronization, these VR-generated marker transitions were applied to the EEG data to define the complete trial epoch from the first target phase to contact with the final target. These epochs were then visually inspected to identify channels or trial epochs containing large nonphysiological artifacts, including ocular, scalp muscle, and movement-related transients. Bad-channel screening was performed during this inspection by checking for channels with persistent excessive noise, saturation, flat-line activity, or large movement-related artifacts. As part of this inspection procedure, MATLAB code automatically flagged epochs containing amplitudes exceeding ±200 μV. Flagged epochs, together with epochs showing large residual nonphysiological artifacts, were subsequently excluded before band-power analysis. Thus, the VR-generated target-contact markers were used to define the EEG segmentation boundaries, whereas the ±200 μV amplitude threshold was applied only after segmentation as a code-assisted criterion for artifact screening. No EEG-based event detection or amplitude threshold was used to determine the segmentation boundaries. Band-power analysis was performed only within the trial window, defined from the onset of participant movement toward the first target to contact with the final target. Trials were analyzed as complete epochs and were not subdivided into individual target-to-target movements. Absolute band power was computed for each selected motor-cortex channel using the MATLAB R2023b ‘bandpower’ function with the default spectral-estimation settings used in the analysis code. Band power was first computed separately for Cz, C1, C2, C3, and C4, and the resulting values were averaged across these five channels to obtain one alpha-band and one beta-band value per trial. Band-limited power was extracted for the alpha (8–12 Hz) and beta (13–30 Hz) frequency bands. Because the present EEG outcomes were limited to sensor-level alpha- and beta-band power, independent component analysis, principal component analysis, source decomposition, and explicit electrocardiographic, ballistocardiograph, or respiratory artifact modeling were not performed. Heart rate (beats per minute) was derived from a PPG signal obtained at the left earlobe by measuring light absorption variations. This measurement was obtained using a Shimmer3 GSR + sensor (Shimmer Research Ltd., Dublin, Ireland) at a sampling rate of 51.2 Hz. All physiological and VR-derived task signals were synchronized offline at the trial level. A single marker sequence generated by the VR task was stored with the recorded dataset. The marker began at its initial value during the first target phase and increased by one after each contact with the currently active target. Following offline synchronization, this common VR-generated marker sequence was used to delineate the corresponding trial and target phases across the EEG, EMG, PPG, and VR-derived data; no independent event markers were generated from the physiological signals. After trial segmentation using these markers, ‘interp’ function in MATLAB R2023b was used to resample the physiological signals so that each trial-level physiological data segment matched the length of the corresponding VR-derived performance data segment. This procedure allowed EMG, EEG, heart-rate/PPG, and VR-derived performance measures to be aligned within the same trial windows before outcome extraction. Because EMG was the only signal used for real-time closed-loop control, its real-time processing pathway is summarized in Figure 2. The offline data-processing workflow used to derive the outcome measures is summarized in Figure 3.

**FIGURE 2:**
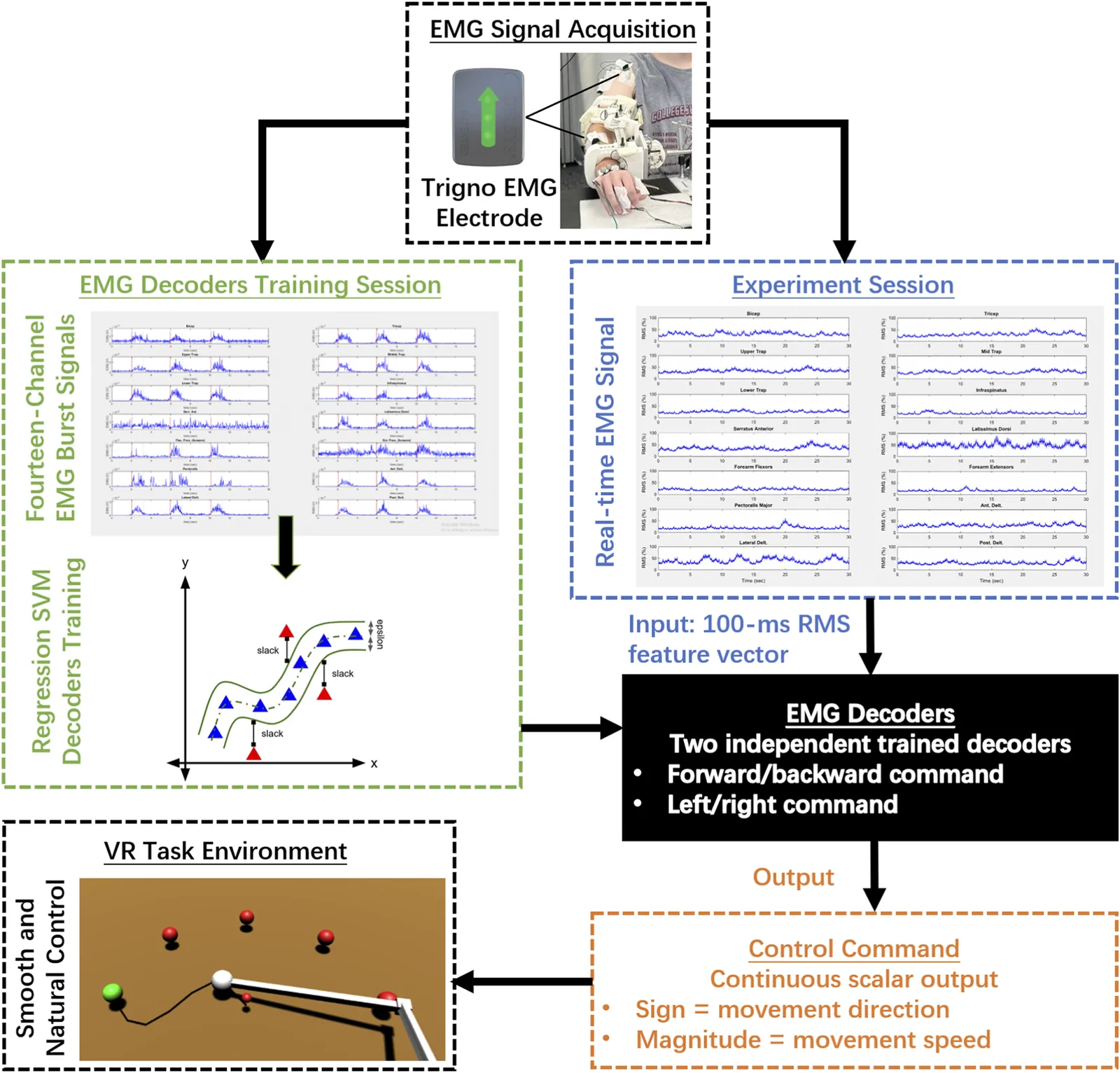
Myoelectric control architecture. Fourteen-channel EMG signals were used to train two SVR decoders and to generate real-time control commands during the experiment. The decoded outputs controlled forward–backward and left–right end-effector movement, enabling smooth, continuous, and natural control of the virtual arm.

**FIGURE 3:**
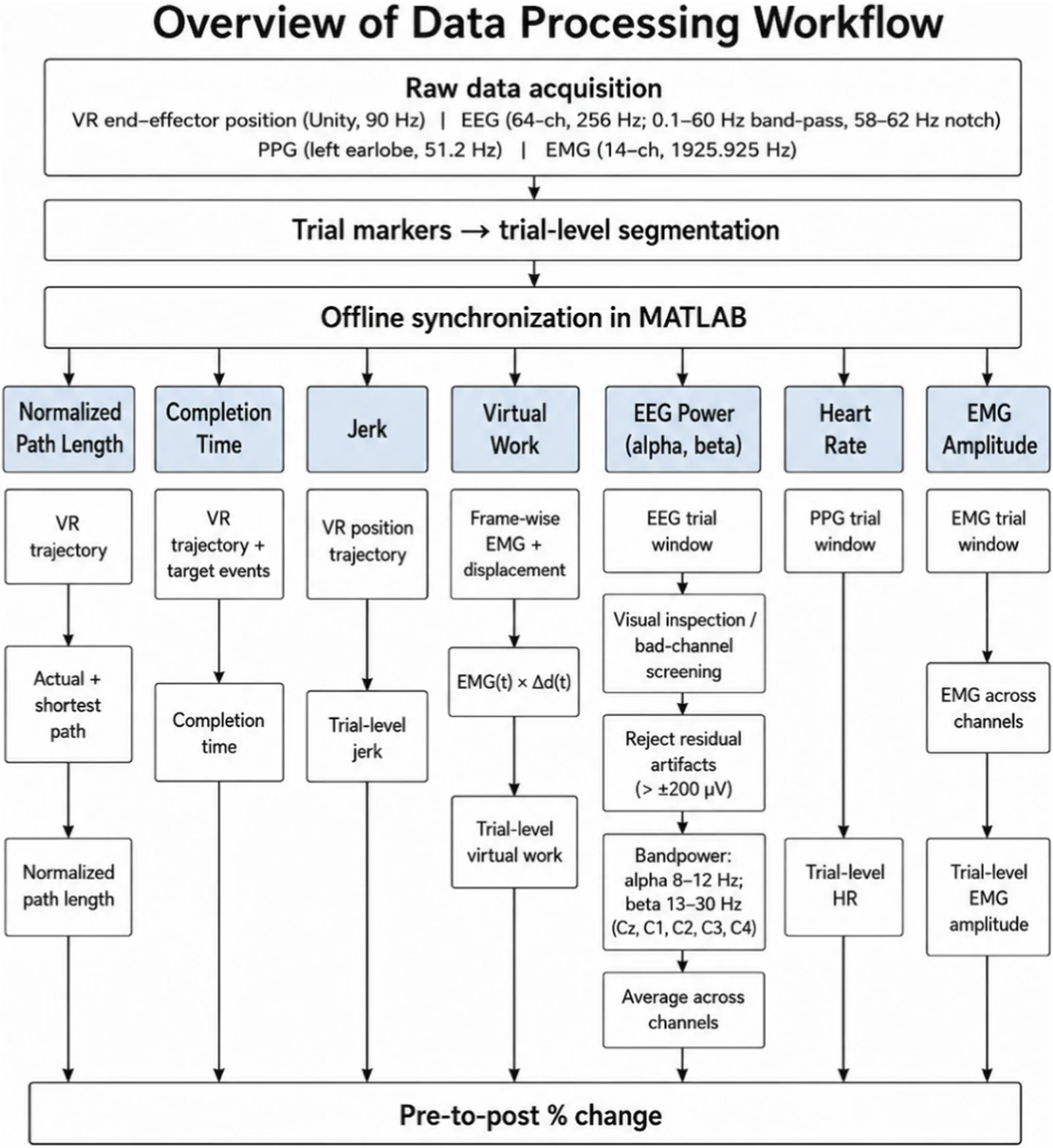
Overview of the offline data-processing workflow. VR-derived and physiological signals were segmented and synchronized at the trial level before normalized path length, completion time, movement jerk, virtual work, EEG power, heart rate, and EMG amplitude were calculated. Pre-to-post percentage changes were then computed for all outcomes.

### 2.4 Customized brace platform

A previously developed VR platform for rehabilitation of upper-limb function (Sanford et al., 2022) was used in this study. The platform was designed to facilitate near-isometric muscle training while incorporating mechanical compliance to support user comfort and reduce potential spasms in clinical populations (Nataraj et al., 2025). The primary structural component was a position-adjustable brace that supported the upper arm against gravity while participants generated semi-isometric muscular efforts for rehabilitative training. This brace system comprises 3D-printed cuffs, hinged and adjustable rods, and a supporting bracket affixed to a table in front of the participant. The arm-mounting assembly secures both the upper arm and forearm in place, whereas an adjustable rod links the arm brace to an auxiliary bracket on the table, permitting participants to position their dominant arms at various angles as prescribed by a physical therapist for training at varied muscle lengths. In this study, participants assumed a comfortable and neutral arm posture, constrained within the following angular ranges: shoulder abduction (45°–75°), shoulder internal rotation (0°–45°), and elbow flexion (90°–120°). A neutral position was defined as the orientation from which participants perceived they could exert substantial forces in four orthogonal directions (forward, backward, left, and right) for commanding movement of a computerized avatar, such as a virtual robotic arm. To provide directional cues, five vibration motors (ERM DC 11000 RPM, Vybronics Inc., China) were strategically placed on the hand of the arm in the brace (Figure 1A) to provide directional cues about displacement errors from target trajectories. Motors were placed on the distal phalanx of the middle finger to cue forward errors, the dorsal aspect of the wrist to cue backward errors, the medial side of the dorsal hand to cue rightward errors, and on the radial (lateral) side of the dorsal hand to cue leftward errors. A marker-based motion capture system (Prime17W infrared cameras, OptiTrack Inc., Corvallis, OR, USA) was used to record the position and orientation of the participant’s reaching hand using four reflective markers tracked by nine infrared cameras. The OptiTrack system was calibrated before data collection according to the manufacturer guidelines to support reliable marker tracking during decoder-training trials. These marker trajectories were used solely to verify the participant’s intended movements during the EMG decoders-training trials (see below) and were not used to compute the primary behavioral or physiological outcome measures. Nevertheless, because this verification step relied on optical tracking, marker occlusion, illumination conditions, and changes in camera–marker geometry could have affected the reliability of movement-intent confirmation during decoder training, although these factors did not directly drive the virtual end effector or determine the reported outcomes. By contrast, wearable inertial measurement units (IMUs) are not dependent on fixed camera line-of-sight or illumination conditions and can provide a more portable alternative for movement monitoring outside controlled laboratory environments (Palazzo et al., 2025; Suglia et al., 2024).

### 2.5 Myoelectric control method: EMG decoders

The overall myoelectric control framework is illustrated in Figure 2. A support vector machine (SVM)-based regression model, an approach widely applied in myoelectric decoding for prosthetics and spinal cord injury rehabilitation, was trained for each participant to convert EMG signals into continuous directional commands for the virtual arm (Liu and Zhou, 2013). A regression-based SVM model was developed using the 'fitrsvm’ function in MATLAB R2023b, with a continuous output formulation chosen to enable smooth, graded control transitions in both movement direction and speed. Before model input, raw EMG from each of the 14 channels was converted into a real-time amplitude feature by computing the root-mean-square (RMS) value over the preceding 100 ms. The resulting 14-channel RMS feature vector was used as the input to both SVM regression models. Two independent models were trained separately: one for the anterior–posterior axis (forward vs. backward) decoding and one for the medial–lateral axis (left vs. right), each producing a continuous scalar output rather than a discrete class label, where the sign indicated movement direction and the magnitude set movement speed. In the VR environment, the 2D command was applied each frame to update the end-effector position as a per-frame displacement, enabling intermediate, smoothly varying velocity. Consequently, greater decoded command magnitude, reflecting stronger or more sustained task-related muscle activation, produced faster end-effector movement.

Decoder-training data were collected during a preliminary set of trials in the VR environment before the experimental conditions (green section in Figure 2). Participants executed exertions within the brace in forward, backward, leftward, and rightward directions at designated levels corresponding to 20% and 40% of their maximum voluntary isometric contraction (MVIC) (Janik et al., 2021). Each exertion was maintained for a 2-s interval, with the central 1-s segment used for training the SVM. The training parameters adhered to those described in (Sanford et al., 2022). Thus, each participant completed eight calibration trials, corresponding to four movement directions tested at two MVIC thresholds, with three exertion bursts collected per trial. The position of the participant’s right hand was monitored via motion capture to ensure the intended directional exertions were achieved during SVM training trials. Using all 14 EMG channels as inputs, model offline training accuracy exceeded 90% for all participants, and participants reported smooth, natural control. Decoder parameters were fixed throughout the experiment across all conditions to avoid confounding performance due to changes in EMG decoding.

### 2.6 VR task environment

A custom VR application was developed in Unity 2019.2.9f1 (Unity Technologies, San Francisco, CA, USA) to enable real-time myoelectric control of a virtual robotic arm for contacting multiple targets. The system ran on a workstation (AMD Ryzen Threadripper 2920X, 12 cores at 3.5 GHz, 32 GB RAM; Windows 10). The robotic end effector was modeled as a sphere attached to a two-link arm whose joint angles were updated via an inverse-kinematics routine (Parger et al., 2018). This inverse-kinematics routine was used only to render the posture of the virtual arm for visual embodiment; participant control, target-contact detection, ASF calculation, and all performance outcomes were based on the two-dimensional end-effector position driven by decoded EMG commands. Therefore, a simplified virtual-arm representation was used to focus attention on end-effector control and to avoid potential mismatch between a generic detailed arm model and individual differences in participants’ arm appearance or proportions. The VR workspace and target positions were constrained within the reachable range of the virtual two-link arm, so the task did not require end-effector positions near singular configurations. Participants viewed the VR scene through an HTC Vive head-mounted display (HTC Corporation, Taiwan), worn over the EEG cap. VR-derived signals (e.g., end-effector kinematics and guidance-cue updates) were logged at 90 Hz. The Unity environment received end-effector position information from MATLAB (MathWorks, Inc., Natick, MA, USA) via an API connection, which also interfaced with MATLAB toolboxes used to train and execute the participant-specific EMG decoders. In the VR coordinate system, both the spherical end effector and the targets were defined as 1 unit in diameter, and the radius from the centered start position to the semicircular array of training targets was set to 7 units.

### 2.7 Individual testing and training trials

As illustrated in Figure 4, the VR interface depicts a target-directed task in which a virtual robotic arm, controlled via myoelectric input, moves within a two-dimensional plane during both testing (Figure 4A) and training trials (Figure 4B). For testing trials, once the trial was initiated (i.e., after the end effector returned to the start position), participants commanded the end effector to contact five randomly arranged targets in an order determined by their own strategy (Figure 4C). All targets were constrained to the same radial distance from the central start position and had the same diameter. Thus, target arrangement varied across testing trials, whereas the start-to-target distance and target size were held constant to reduce systematic differences in movement demand across target positions.

**FIGURE 4:**
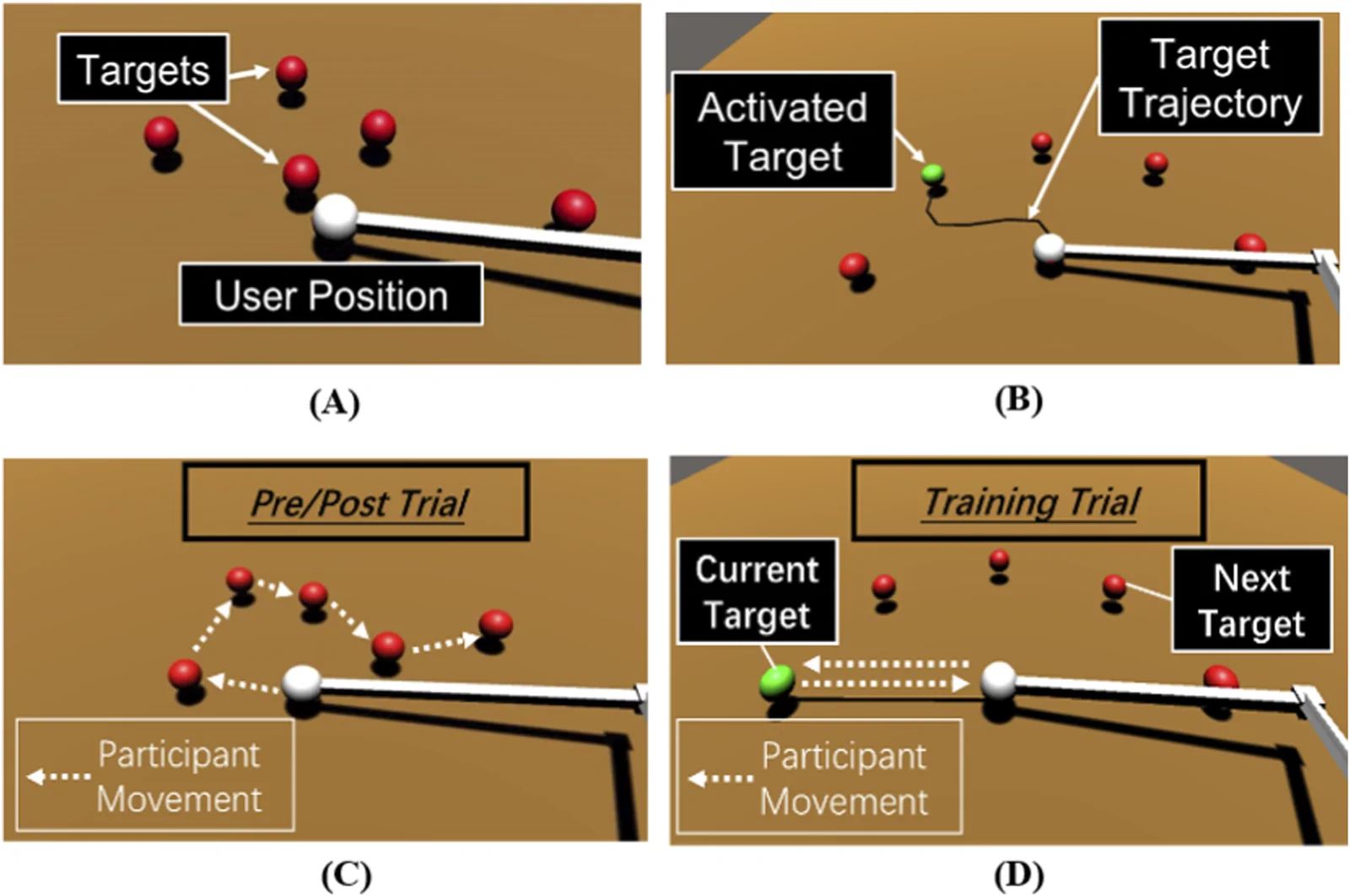
VR task environment during testing and training blocks. **(A)** Pre- and post-training testing environment, in which participants controlled the virtual robotic arm end effector to contact five targets. **(B)** Training environment, in which participants moved toward the currently activated target. **(C)** Schematic of the pre/post-training task. **(D)** Schematic of the straight-line training task.

During training trials (Figure 4D), the five targets were positioned at fixed initial locations along a semicircle, evenly distributed between 0° and 180°. The end effector began at a centrally located, equidistant starting point, from which participants were required to move toward the currently active target (indicated by a color change to green whereas the remaining targets remained red). Participants sequentially contacted each cued target until all five targets had been reached. The target-activation order was not disclosed to participants in advance. Participants were instructed to follow the displayed target trajectory (black straight-line or sinusoidal curve) as closely as possible while approaching each target.

### 2.8 Experimental protocols

Each participant completed one experimental session lasting 3–4 h, which included debriefing, equipment setup, EMG decoding procedures, and completion of trial blocks across the four ASF training conditions. The setup period (∼30 min) involved positioning and fitting the brace (including participant-specific padding adjustments) and placing skin-surface sensors for physiological recordings. Participants then completed several practice trials to become familiar with the myoelectric control of the avatar. The remainder of the session (∼2–3 h) was dedicated to evaluating training effects across the four experimental conditions. For each condition, participants completed three consecutive trial blocks: (1) a pre-training block consisting of five testing trials (five randomly arranged targets) performed without ASF to establish baseline values for all measures; (2) a training block consisting of 10 training trials in which ASF was provided while contacting five targets arranged along a semicircle; and (3) a post-training block that repeated the same five testing trials to quantify training-related changes relative to baseline across all measures. On average, testing and training trials required approximately 10 s and 40 s, respectively. These durations refer to the different trial types rather than to the straight-line versus sinusoidal difficulty conditions: testing trials were used for no-ASF pre-to-post assessment, whereas training trials involved repeated trajectory tracking under the assigned ASF–difficulty condition. The order of the four ASF–difficulty conditions was randomized across participants and arranged to be approximately balanced across the sample to reduce systematic sequence effects. In addition, 5–10 min rest breaks were provided between conditions to reduce fatigue accumulation, consistent with fatigue-control considerations in repeated upper-limb motor-task protocols (Suglia et al., 2026). No separate washout block was included between experimental conditions. The post-training block was performed without ASF to quantify short-term training-related changes relative to the pre-training baseline, rather than to remove residual effects. Each condition began with its own no-ASF pre-training block, which served as the condition-specific baseline for pre-to-post comparisons. For clarity, ASF refers to the general category of augmented sensory feedback, whereas VF and VHF denote the two ASF modalities tested in this study: visual feedback alone and combined visual–haptic feedback, respectively. The four experimental conditions were as follows.

1. VF straight-line: Participants were required to command the end effector from the central start position toward the designated target while following a black straight-line trajectory (Figure 5A). When the end effector deviated from the prescribed trajectory beyond a threshold (0.25 units in each direction), a guidance sphere appeared at the point on the target trajectory corresponding to the current position of the end effector (i.e., the projection of the current position onto the trajectory). The visual salience of the sphere scaled with the magnitude of deviation: its opacity increased as the offset grew, reaching full opacity (i.e., fully solid) once the deviation exceeded two units (Figure 5C).
2. VF sinusoid: Participants were instructed to drive the end effector from the central start position toward the designated target while tracking a black sinusoidal trajectory (Figure 5B). During these trials, participants received visual-only ASF, implemented in the same manner as described above.
3. VHF straight-line: Participants were instructed to move the end effector from the central start position toward the designated target while tracking a black straight-line trajectory. In addition to the visual ASF described above, haptic ASF was provided concurrently (Figure 5D). A set of vibration motors placed on the hand conveyed corrective cues analogous to those encoded by the guidance sphere, with the intent of signaling deviations from the target trajectory and prompting online correction. Specifically, the actuator corresponding to a given direction was activated to indicate that the participant should move in that direction to return toward the prescribed trajectory. Vibrotactile stimulation was delivered at four discrete frequency levels that increased monotonically with deviation magnitude, spanning the bandwidth from 0.25 units of deviation (lowest frequency) to 2 units of deviation (highest frequency).
4. VHF sinusoid: Participants were instructed to drive the end effector from the central start position toward the designated target while tracking a black sinusoidal trajectory and receiving combined visual and haptic ASF.

**FIGURE 5:**
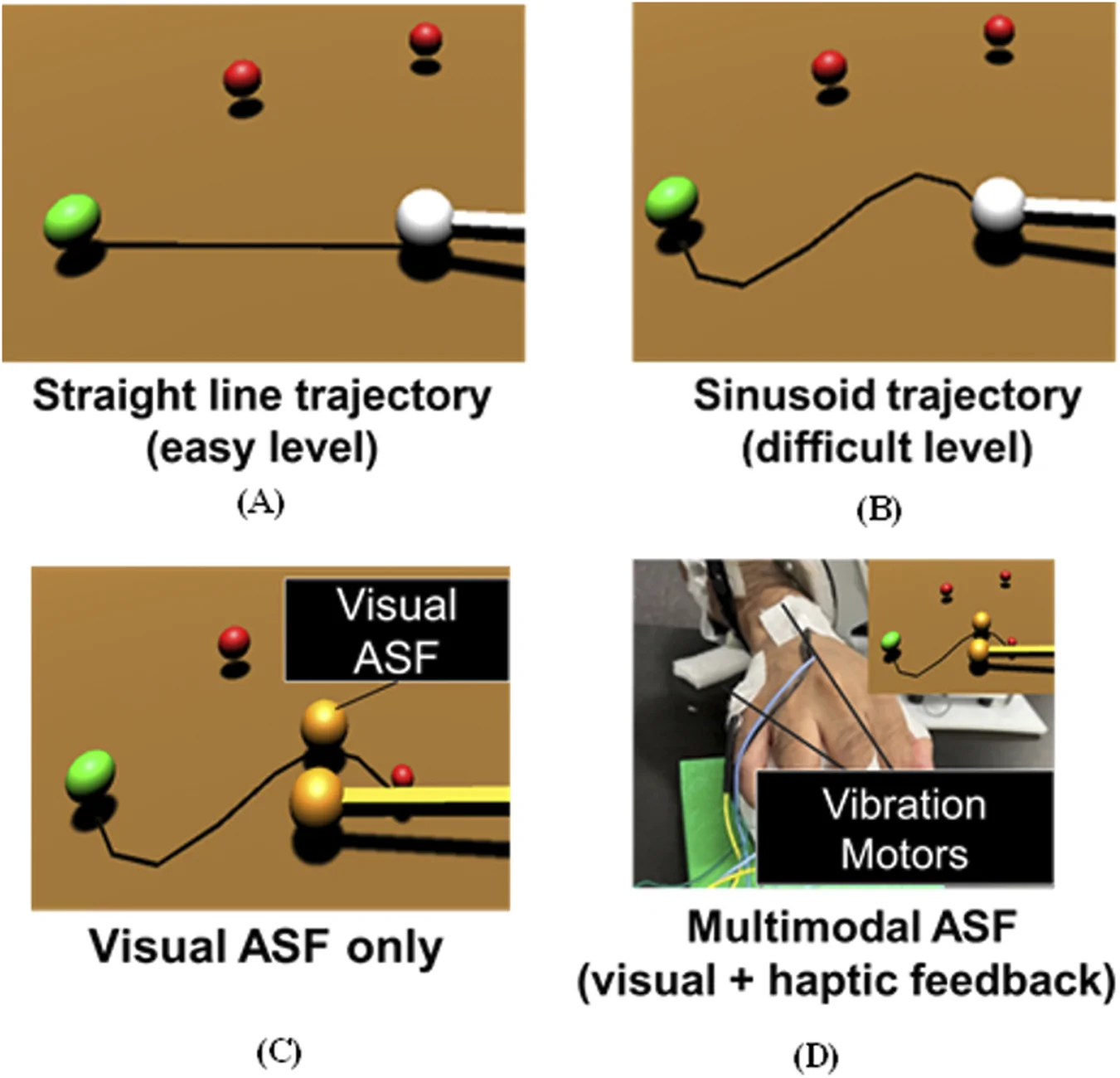
Two training elements (task difficulty level and ASF modality) consisting of four experimental conditions. **(A)** easier task difficulty level when the target trajectory is a straight-line. **(B)** more difficult task difficulty level when the target trajectory is a sinusoid. **(C)** visual ASF only when deviation occurs. **(D)** multimodal ASF: haptic feedback (provided by vibration motors) combined with visual feedback.

After each condition, participants completed a brief perceptual survey to evaluate their experience with the training modality across three aspects: sense of agency (Moore and Obhi, 2012), motivation, and perceived utility. Each aspect was assessed with a single statement rated on a 0–100 continuous scale, where higher scores indicated stronger agreement with the statement. The statements were.

1. I was in full control of virtual arm during training.
2. The mode of training was motivating.
3. The mode of training was useful for improving performance.

### 2.9 Analysis

The offline data-processing workflow used to derive the behavioral and physiological outcome measures is summarized in Figure 5. All statistical analyses were performed in MATLAB R2023b. Physiological responses and motor performance in the post-training block were compared with results from the pre-training block to characterize the effects of the training elements. Motor performance was assessed by two metrics: normalized path length (the ratio of the actual path length to the shortest possible path) and task completion time, both of which are well-established and valid indicators of movement performance (Schwarz et al., 2019). Movement smoothness was additionally quantified using jerk derived from the VR end-effector trajectory. For each trial, jerk was computed from the time-resolved position signal and summarized as a trial-level result, with larger values indicating less smooth movement and greater abrupt correction. Physiological measures included EEG (alpha and beta band power) from the motor cortex, heart rate (beats per minute), and EMG amplitude.

EEG band power was quantified using the ‘bandpower’ function (MATLAB R2023b) for the alpha (8–12 Hz) and beta (13–30 Hz) frequency bands. The underlying computation can be expressed as

$$P_{\text{band}}=\frac{1}{f_{2}-f_{1}}\int_{f_{1}}^{f_{2}}S_{xx}\left(f\right)df$$

where $\left[f_{1},f_{2}\right]$ denotes the frequency bounds of the band of interest and $S_{xx}\left(f\right)$ is the power spectral density (PSD) of the signal. EMG amplitude was characterized as trial-level root-mean-square (RMS) activity across the recorded EMG channels within each complete movement epoch and was used as a relative measure of task-related myoelectric activity, defined as

$$A_{\text{EMG}}=\sqrt{\frac{1}{N}\sum_{n=1}^{N}|x_{n}{|}^{2}}$$

where $x_{n}$ is the EMG signal at sample n and N is the total number of samples. To characterize effort-related task cost, the authors introduced an EMG-displacement–based virtual work metric. For each frame k , a frame-wise virtual work increment was computed as the product of the EMG amplitude at that frame, $A_{\text{EMG}}\left(k\right)$ , and the magnitude of the corresponding end-effector displacement increment,

$$∥\Delta x\left(k\right)∥$$

where

$$\Delta x\left(k\right)=x\left(k+1\right)‐x\left(k\right)$$

Trial-level virtual work was then obtained by summing these frame-wise increments across the entire movement. Hence, the virtual work computation can be defined as:

$$Wv=\sum_{k=1}^{N-1}A_{EMG}\left(k\right)∥x\left(k+1\right)-x\left(k\right)∥$$

Because external force and joint torque were not directly measured, and because virtual displacement was generated from decoded EMG commands, this metric was not interpreted as direct mechanical work or as an outcome independent of the myoelectric control architecture. Instead, virtual work was used as an effort-related task-cost metric that combined myoelectric activity with the amount of virtual movement produced during the task. Accordingly, virtual work was interpreted alongside normalized path length, completion time, jerk, and EMG amplitude when evaluating motor-control efficiency.

A *post hoc* analysis of motor performance and physiological measures was conducted including all 14 participants. To quantify training effects, all results were calculated from each block of pre-training and post-training trials and used to compute percentage change as $\left(\frac{post-pre}{pre}\right)\times 100\%$ . Consequently, a positive percentage change denotes an increase in the underlying metric (e.g., longer paths, longer times, larger jerk, higher power amplitude). In contrast, negative values denote decrease after training. Because EMG signals served as the control input under fixed decoder parameters, changes in RMS EMG were interpreted as a measure of neuromuscular command effort, where decreases suggest more economical motor control and increases suggest greater activation demand or more frequent corrective commands. Accordingly, EMG amplitude was interpreted together with path length, completion time, jerk, heart rate, and virtual work rather than as an isolated physiological-load measure.

Data were inspected at the individual trial level. For all metrics, data within each trial were visually inspected in time order to identify obvious artifacts or contamination attributable to non-physiological factors (e.g., poor electrode–scalp coupling in the EEG cap). Trials exhibiting clear contamination were manually flagged and excluded from subsequent analyses. Outliers for all metrics were identified with MATLAB R2023b ‘rmoutliers’ function with a ±2 SD threshold, where SD was computed independently within each experimental condition. Across the full exclusion procedure, 21.9% of trials were removed, and exclusions did not cluster in any single condition. Normality of the retained data was assessed using MATLAB R2023b ‘lillietest’ function, and all datasets used for inferential testing satisfied the normality criterion. Two-way repeated-measures ANOVA was applied to evaluate the effects of ASF modality, task difficulty, and their interaction on each outcome measure.

The two-way repeated-measures ANOVA was treated as the primary inferential analysis because it directly tested the main effects of ASF modality and task difficulty, as well as their interaction. To complement this factorial analysis, a secondary one-way repeated-measures ANOVA was also conducted by treating the four ASF–difficulty combinations as four levels of a single within-subject factor. This follow-up analysis was used to evaluate whether the complete training conditions differed at the level of the actual intervention prescriptions experienced by participants. Thus, the one-way ANOVA provided a condition-level omnibus test, whereas the two-way ANOVA provided factor-level interpretation. Significant condition-level effects were further examined using *post hoc* pairwise comparisons to identify specific differences between training configurations. Correction was done for the *post hoc* pairwise comparisons using Tukey-Kramer adjustment in ‘multcompare’ function in MATLAB R2023b. The primary 2 × 2 ANOVA was the planned factorial analysis. Given the modest sample size, a *post hoc* sensitivity power analysis was conducted for normalized path length and completion time, which were the primary performance-related outcomes used to evaluate training-related behavioral change. For the 2 × 2 within-subject design with N = 14, α = 0.05, and target power = 0.80, each main effect and interaction can be represented as a one-degree-of-freedom within-subject contrast. The sensitivity analysis indicated that the study was positioned to detect standardized within-subject contrast effects of approximately dz ≥ 0.81. Therefore, the analysis was sufficiently sensitive to detect large effects in the primary performance outcomes, whereas smaller ASF-modality, task-difficulty, or interaction effects cannot be excluded.

In addition, this study introduced an exploratory composite “physiological index” to summarize the overall magnitude of physiological change following training. For all 14 participants, this index was computed as the sum of absolute pre-to-post percentage changes in EEG power, EMG amplitude, and heart rate, rather than from raw physiological signal values. Thus, each component was first expressed as a percentage change from its own pre-training value. Because the index combines heterogeneous physiological measures without z-normalization or weighting, it was interpreted only as a descriptive summary of overall physiological modulation, not as a normalized physiological score or a standalone indicator of beneficial or unfavorable response. Individual EEG, EMG, and heart-rate outcomes were also reported separately and interpreted alongside the behavioral and task-cost measures. Two-way ANOVA was applied to evaluate the effects of the individual factors on each outcome measure.

For exploratory analyses, a “best-case” training condition was identified for each participant as the condition producing the largest reduction in normalized path length. The ordinal position of this condition within each participant’s randomized four-condition sequence was also recorded to determine whether best-case performance occurred disproportionately early or late in the experimental session. The numbers of participants whose best-case condition occurred in the first, second, third, or fourth sequence position were summarized descriptively. Percentage changes in each metric under this best-case condition were compared with the mean of the remaining three conditions using paired t-tests. Because the best-case condition was defined as the condition producing the largest reduction in normalized path length, the paired comparison for path length is circular by construction and is reported for completeness rather than as an inferential result. Outcomes that covary with path length may likewise show inflated differences under this selection. The p-values in Table 1 are reported without correction across outcomes and are interpreted as exploratory descriptions of the best-case profile rather than as confirmatory tests.

**TABLE 1:** Exploratory paired t-test p-values comparing each participant’s best-case condition with the mean of the remaining three conditions.

Measurement	p value
Normalized path length	**6.37e-07**
Completion time	**0.035**
Jerk	0.315
Alpha band power	0.313
Beta band power	**0.039**
Heart rate	**0.040**
EMG amplitude	**0.014**
Virtual work	**0.0387**

bolded values indicate *p* < 0.05.

The best-case condition was selected as the condition yielding the largest reduction in normalized path length; the path-length comparison is therefore circular by construction and is shown for completeness only. Values are uncorrected across outcomes.

To examine relationships between motor performance and physiological responses, a simple linear regression analysis was performed between the post-training change in path length and the corresponding changes for completion time and mean EMG amplitude, pooling data from all four training conditions. These analyses were exploratory and were used to summarize descriptive associations rather than to establish predictive or causal relationships. To address the modest sample size and the possibility of non-normal or non-linear associations, Spearman’s rank-order correlation coefficients (ρ) were also computed as a complementary nonparametric analysis.

## 3 Results

The resulting ANOVA p-values and partial eta-squared effect sizes (ηp^2^) for all major metrics for factors of ASF modality and difficulty level are summarized in Table 2. Partial eta-squared was used to indicate the magnitude of each main effect and interaction in addition to statistical significance. Table 1 shows the paired t-test values that compared participants’ best case with mean of remaining three conditions for all major metrics.

**TABLE 2:** Two-way repeated-measures ANOVA results for the main effects of ASF modality, task difficulty, and their interaction.

Measurement	ASF modality	Difficulty level	Interaction
Normalized path length	**p = 0.037** ηp^2^ = 0.29	p = 0.58 ηp^2^ = 0.024	p = 0.43 ηp^2^ = 0.049
Completion time	**p = 0.0035** ηp^2^ = 0.49	p = 0.89 ηp^2^ = 0.0015	p = 0.93 ηp^2^ = 0.001
Jerk	**p = 0.048** ηp^2^ = 0.27	p = 0.45 ηp^2^ = 0.045	p = 0.079 ηp^2^ = 0.22
Alpha band power	p = 0.12 ηp^2^ = 0.18	p = 0.79 ηp^2^ = 0.006	p = 0.86 ηp^2^ = 0.003
Beta band power	p = 0.14 ηp^2^ = 0.16	**p = 0.040** ηp^2^ = 0.29	p = 0.14 ηp^2^ = 0.16
Heart rate	p = 0.27 ηp^2^ = 0.093	p = 0.86 ηp^2^ = 0.0025	p = 0.095 ηp^2^ = 0.21
EMG amplitude	**p = 0.024** ηp^2^ = 0.33	p = 0.41 ηp^2^ = 0.053	p = 0.42 ηp^2^ = 0.051
Virtual work	**p = 0.049** ηp^2^ = 0.27	p = 0.22 ηp^2^ = 0.11	p = 0.81 ηp^2^ = 0.005
Physiological index	p = 0.52 ηp^2^ = 0.033	**p = 0.047** ηp^2^ = 0.27	p = 0.15 ηp^2^ = 0.15

bolded values indicate *p* < 0.05. ηp^2^ = partial eta squared.

Each cell reports the p-value and partial eta-squared effect size (ηp^2^).

### 3.1 Behavioral and physiological effects of training conditions

Group-mean percentage changes in movement-related outcomes are shown in Figure 6. Across both metrics, the multimodal straight-line condition (VHF straight-line) yielded the largest reductions (−4.69% in path length, −6.12% in completion time), whereas VF straight-line increased path length and completion time; the sinusoid conditions produced more modest changes. These patterns are reflected in significant main effects of feedback modality for path length (p = 0.037) and completion time (p = 0.0035), with no main effect of difficulty or interaction (Table 2). Post hoc pairwise comparisons following the condition-level one-way repeated-measures ANOVA showed significant differences between VF straight-line and VHF straight-line for both normalized path length (p = 0.0479) and completion time (p = 0.0484).

**FIGURE 6:**
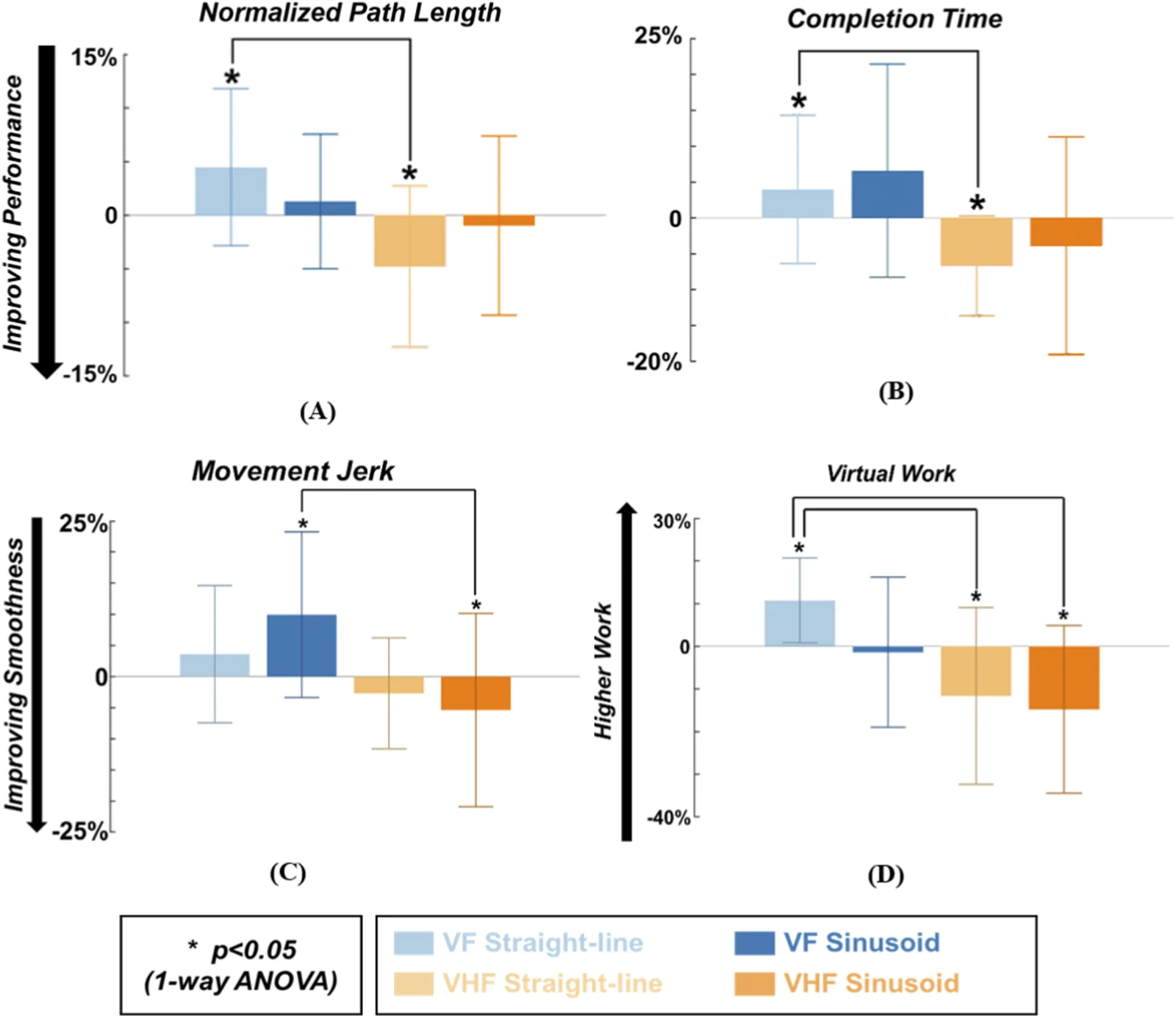
Group-mean pre-to-post percentage changes in motor performance, movement smoothness, and effort-related task cost across the four ASF–difficulty training conditions. **(A)** Normalized path length. **(B)** Completion time. **(C)** Movement jerk. **(D)** EMG-displacement–based virtual work. Negative values indicate reductions from pre-to post-training. Asterisks/brackets indicate significant *post hoc* pairwise differences (p < 0.05) following the condition-level one-way repeated-measures ANOVA.

Changes in movement jerk (Figure 6C) showed a different pattern from the primary performance metrics shown in Figures 6A,B. Jerk increased under both VF conditions, with the largest increase observed in VF sinusoid (+9.94%), whereas both VHF conditions showed reductions, with the largest decrease in VHF sinusoid (−5.67%). A significant pairwise difference was observed between VF sinusoid and VHF sinusoid (p = 0.026), with the latter showing greater movement smoothness post-training. Consistent with the performance findings, ASF modality was the primary source of variance for movement jerk (two-way ANOVA: p = 0.048), with task difficulty and the interaction term both non-significant. Pre-to-post changes in virtual work are shown in Figure 6D. Virtual work increased following VF straight-line training (+10.75%) and remained near baseline for VF sinusoid, whereas both VHF conditions showed post-training reductions, most prominently under VHF sinusoid (−13.33%). Post hoc pairwise comparisons confirmed significant differences between VF straight-line and both VHF conditions (p = 0.015). Two-way ANOVA yielded a significant main effect of ASF modality (p = 0.049), with no significant effect of task difficulty (p = 0.22) or their interaction.

Pre-to-post percentage changes in cortical and peripheral physiological responses are shown in Figure 7. Alpha-band power (Figure 7A) showed a marked post-training increase under VF sinusoid (+18.72%), which differed significantly from both VHF conditions (p = 0.020); however, two-way ANOVA revealed no significant main effects of ASF modality (p = 0.12), task difficulty (p = 0.79), or their interaction. In contrast, beta-band power (Figure 7B) was significantly modulated by task difficulty (p = 0.040), with VHF sinusoid producing the largest decrease and VF straight-line showing an increasing trend; a significant pairwise difference was observed between these two conditions (p = 0.030). ASF modality (p = 0.14) and the interaction term (p = 0.14) did not reach significance. Because increases or decreases in EEG band power do not directly indicate a uniformly improved or worsened physiological state, these EEG changes were interpreted in the Discussion in relation to behavioral performance, movement smoothness, heart rate, EMG amplitude, and virtual work.

**FIGURE 7:**
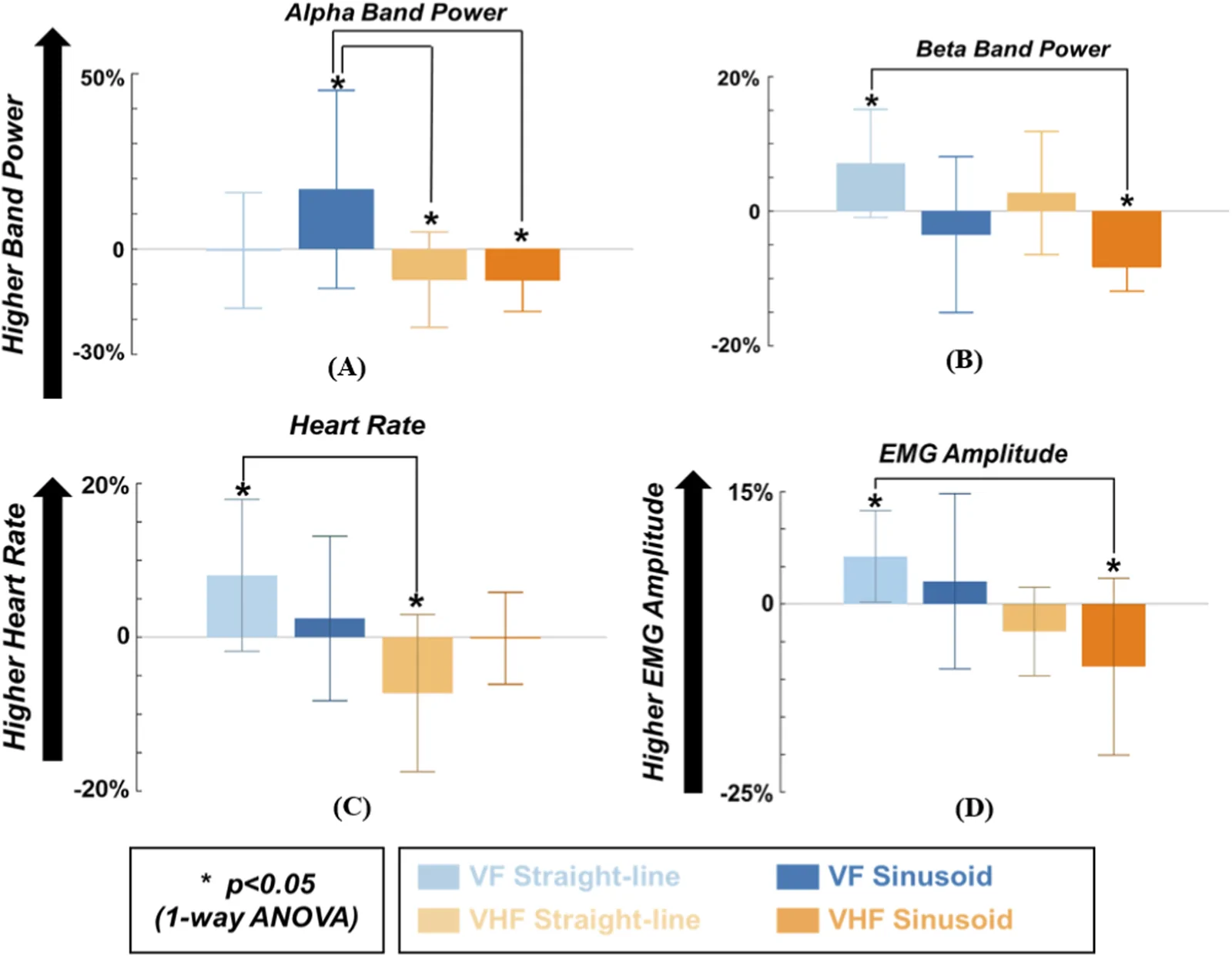
Group-mean pre-to-post percentage changes in physiological responses. **(A)** Alpha-band power. **(B)** Beta-band power. **(C)** Heart rate. **(D)** EMG amplitude.

For heart rate (Figure 7C), multimodal feedback in the straight-line task decreased heart rate, whereas VF in the same task increased it; only the contrast between VF straight-line and VHF straight-line reached significance (p = 0.044), and no significant main effects were found in the two-way ANOVA (feedback p = 0.27; difficulty p = 0.86). EMG amplitude across all muscles (Figure 7D) tended to increase under visual feedback and decrease under multimodal feedback. A significant main effect of feedback modality was observed (p = 0.024), and pairwise comparison showed a significant difference between VF straight-line and VHF sinusoid (p = 0.0168).

The physiological index results are shown in Figure 8, with the absolute percentage change for each measurement annotated directly on the plot. The two sinusoidal (higher-difficulty) conditions, VF–sinusoid and VHF sinusoid, were associated with greater overall physiological perturbation, as reflected by higher composite physiological index values. The two-way ANOVA indicated a significant main effect of task difficulty on the composite physiological index (p = 0.047), whereas neither ASF modality (p = 0.52) nor the interaction (p = 0.15) showed a significant effect.

**FIGURE 8:**
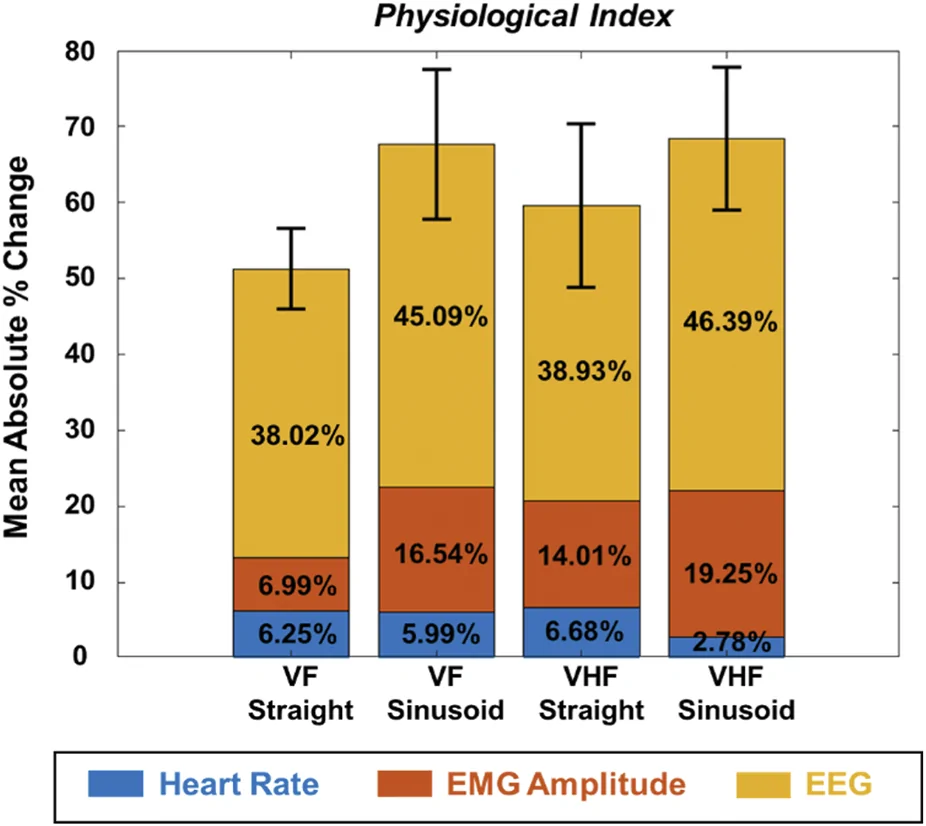
Physiological index: sum of the absolute percentage changes after training in physiological states (EEG, EMG, and HR) across the four training conditions.

### 3.2 Perceptual responses and participant-specific best-case conditions

Perceptual survey responses are shown in Figure 9. For the sense of agency, VF straight-line and VHF sinusoid elicited similarly high ratings. Motivation and utility exhibited similar patterns: VHF sinusoid received the highest endorsement, followed by VHF straight-line. Among the visual-only conditions, VF straight-line received marginally higher motivation ratings than VF sinusoid, whereas the reverse pattern was observed for perceived utility. Collectively, VHF was favored over VF in subjective experience, and pairing VHF with the higher-difficulty sinusoid task yielded the most positive ratings.

**FIGURE 9:**
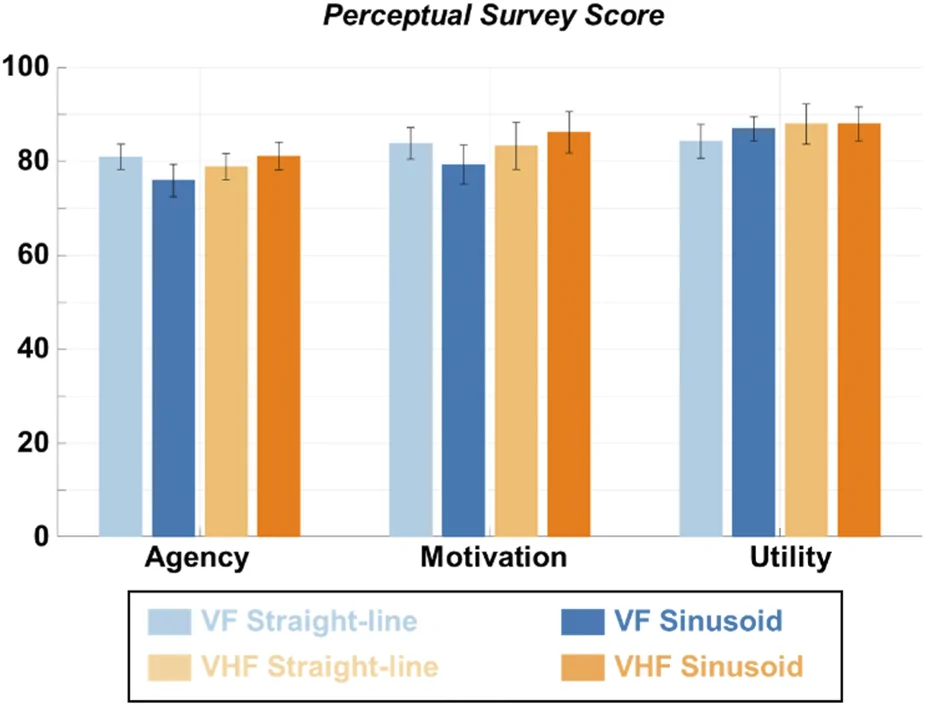
Perceptual survey score (taken after the completion of each condition). Higher score reflects more agreement towards three statements in the survey.

Figure 10 presents an exploratory comparison between each participant’s best-case condition and the mean of the remaining three conditions. Excluding the circular path-length comparison, uncorrected p-values below 0.05 were observed for completion time, beta-band power, heart rate, EMG amplitude, and virtual work, whereas movement jerk (p = 0.315) and EEG alpha-band power (p = 0.313) did not meet this threshold (Table 1). These comparisons are interpreted descriptively because the best-case condition was selected and evaluated using the same data, and differences in outcomes that covary with path length may be inflated by this selection procedure. Best-case conditions were distributed across all four training combinations: VF straight-line (n = 4), VF sinusoid (n = 1), VHF straight-line (n = 5), and VHF sinusoid (n = 4). With respect to experimental sequence, the best-case condition occurred in the first, second, third, and fourth sequence positions for 2, 2, 6, and 4 participants, respectively.

**FIGURE 10:**
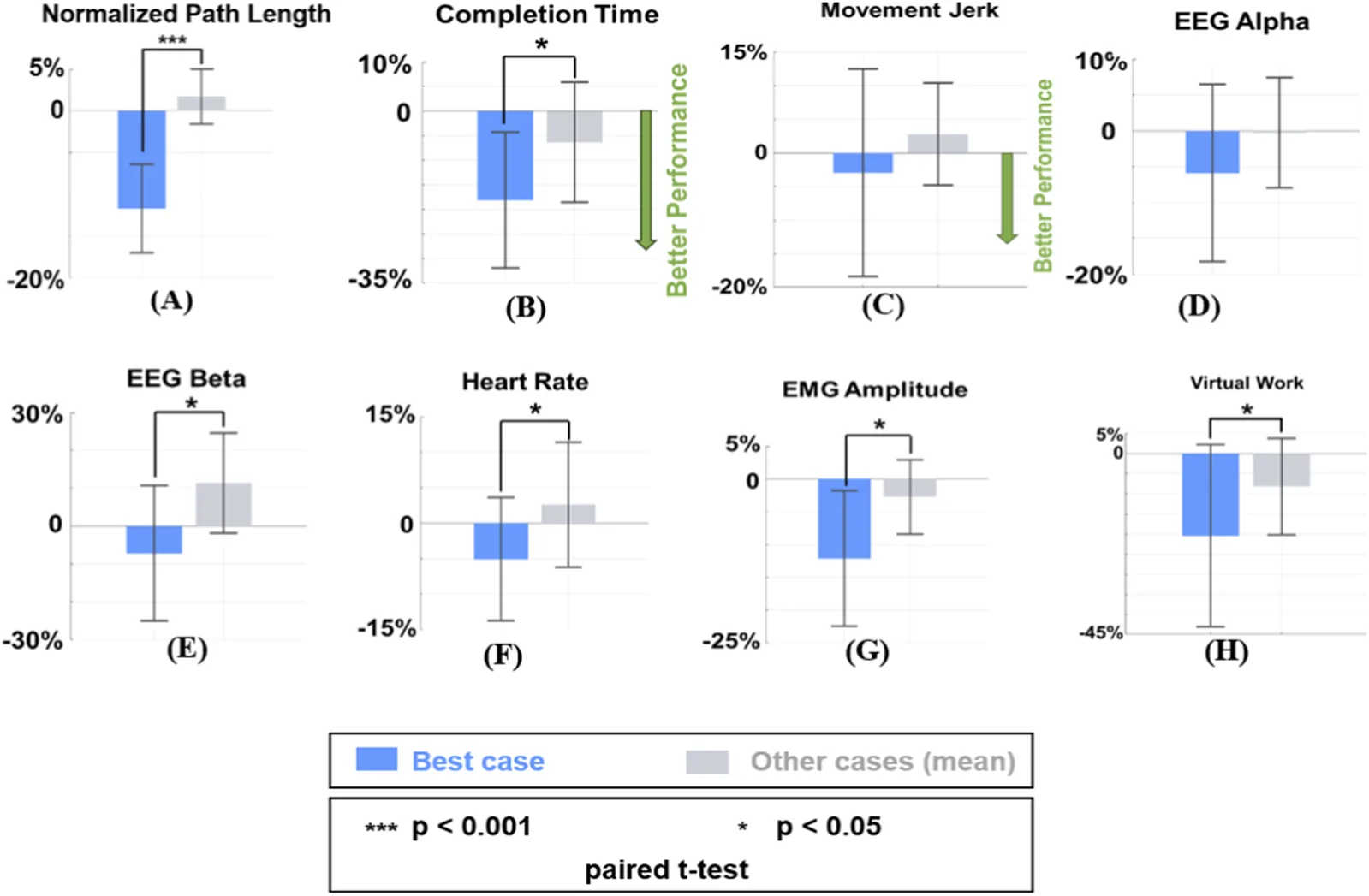
Exploratory comparison of pre-to-post percentage changes for each participant’s best-case condition (blue) and the mean of the remaining three conditions (gray). The best-case condition was selected as the condition yielding the largest reduction in normalized path length; therefore, the path-length panel is circular by construction and is shown for completeness only. Panels **(A)** normalized path length **(B)** completion time **(C)** movement jerk **(D)** EEG alpha-band power **(E)** EEG beta-band power **(F)** heart rate **(G)** EMG amplitude; and **(H)** virtual work.

### 3.3 Relationships among behavioral and physiological changes

Relationships between post-training changes in normalized path length and changes in completion time or EMG amplitude are shown in Figure 11. Normalized path length change was positively associated with completion time change using the linear regression fit shown in the figure (slope = 0.818, p = 1.48 × 10^−4^), and this relationship was also supported by the complementary Spearman correlation analysis (ρ = 0.298, p = 0.0221). Data points with greater reductions in path length generally showed greater reductions in completion time, whereas data points with increased path length tended to show increased completion time. Normalized path length change was also positively associated with EMG amplitude change using the linear fit (slope = 0.918, p = 0.00126), with the complementary Spearman analysis showing a similar positive association (ρ = 0.431, p = 0.000741).

**FIGURE 11:**
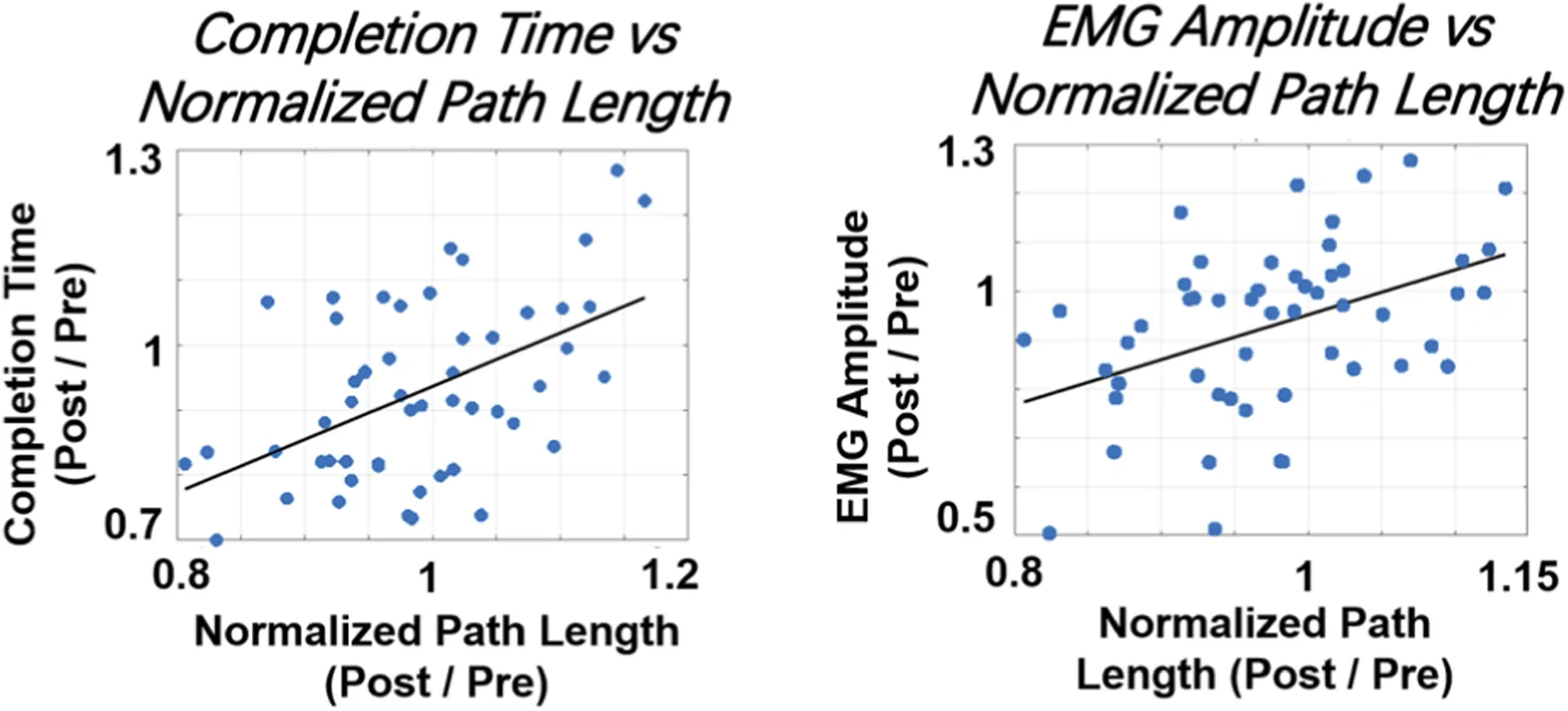
Primary performance metric of path length (x-axis) versus other measures (y-axis) across participant-level means over the four training conditions. Values are expressed as changes after training. Panels show scatter plots and linear regression fits for path length versus completion time (left) and EMG amplitude (right).

## 4 Discussion

This study examined how ASF modality (unimodal visual vs. multimodal visual–haptic) and task difficulty (straight-line vs. sinusoidal trajectories) jointly shaped short-term motor training outcomes within a VR-based upper-extremity rehabilitation platform. Training effects were assessed across multiple complementary domains: motor performance and movement quality, cortical and peripheral physiological responses, effort-related task cost, and subjective perceptual experience. The findings are interpreted in light of two guiding principles: that multimodal feedback can accelerate motor learning by simultaneously engaging multiple sensory channels (Sigrist et al., 2013), and that excessive task or feedback demands may impede learning by overtaxing attentional resources, both of which were reflected in the observed patterns across training conditions. Accordingly, the following discussion considers not only whether training improved performance, but also whether those changes were accompanied by smoother and more economical movement execution, and then relates these patterns to neural, peripheral physiological (HR and EMG), and participant-specific responses relevant to personalized training design.

Motor performance results suggest that ASF modality, rather than task difficulty, was the dominant determinant of group-level behavioral gain. Multimodal feedback, particularly in the straight-line condition, was associated with the clearest post-training reductions in normalized path length and completion time, whereas visual-only feedback under the same difficulty level was associated with increases in both measures, a divergence suggesting that feedback modality, rather than task structure, was the dominant driver of behavioral outcome. The sinusoid conditions yielded comparatively modest changes overall, indicating that increased trajectory complexity alone did not strongly alter short-term behavioral improvement at the group level. These findings suggest that the type of sensory guidance plays a primary role in determining short-term behavioral gains, at least at the group level. This is consistent with the view that concurrent haptic and visual cues provide richer, redundant error information that facilitates more targeted online movement correction.

Jerk provided a complementary view of training effects by indexing movement smoothness rather than task-level success alone. The jerk results suggest a modality-dependent difference in how movements were executed after training: visual-only feedback, particularly under the more demanding sinusoidal trajectory, was associated with increased post-training jerk, whereas multimodal feedback consistently reduced jerk, with the most pronounced decrease observed under VHF sinusoid. This pattern suggests that, under the elevated control demands imposed by sinusoidal curvature, multimodal guidance facilitated more continuous and anticipatory trajectory correction, thereby reducing the abrupt, reactive adjustments that manifest as high jerk. In contrast, visual-only cues under the same conditions may have been insufficient to prevent reactive correction strategies, resulting in less smooth movement execution. Accordingly, the influence of ASF modality extended beyond overall task outcome to the quality of movement execution itself, which provides an important context for interpreting the accompanying changes in physiological and effort-related cost measures.

Beyond movement kinematics, the two feedback modalities were associated with markedly different patterns of peripheral physiological response, spanning cardiovascular activity, neuromuscular command effort, and effort-related task cost. In this context, heart rate was interpreted as a supportive marker of autonomic arousal and cardiovascular task demand, rather than as a direct measure of motor performance. The heart-rate decrease observed during VHF straight-line training may reflect the lower trajectory complexity and reduced need for sustained corrective control in this condition, and may also partly reflect acclimatization or recovery during the repeated VR task. Multimodal ASF was associated with a convergent pattern of benefit: improved task performance and smoother movement were accompanied by lower peripheral physiological load, as reflected by reductions in both heart rate and EMG amplitude. In contrast, visual-only ASF, particularly in the straight-line condition, showed a less favorable pattern, with poorer task-level improvement accompanied by higher heart rate and EMG amplitude, indicating greater peripheral physiological burden and effort demand. Virtual work followed a closely parallel pattern: conditions yielding better performance and lower physiological burden also incurred less neuromuscular effort-displacement cost, further reinforcing the interpretation that multimodal feedback enabled more efficient motor execution overall. Collectively, these patterns indicate that ASF modality shaped not only task-level motor outcomes but also the physiological and effort-related costs of movement execution. Multimodal guidance was associated with a more favorable profile, improved performance alongside reduced peripheral load and task cost, whereas visual-only guidance, even in the less complex straight-line condition, was linked to poorer performance concurrent with elevated heart rate, EMG amplitude, and virtual work. This dissociation has potential implications for training sustainability, as conditions that impose high physiological demand without commensurate performance gains may be less suitable for extended rehabilitation use.

EEG patterns provided a neural context for the modality-dependent performance–cost coupling described above. Across conditions, alpha power tended to decrease after VHF across difficulty levels, whereas VF paired with the more demanding sinusoidal task showed an opposite (increasing) trend; beta power was more strongly shaped by difficulty, with a larger reduction under VHF sinusoid and an increasing trend under VF straight-line. This alpha–beta dissociation suggests that the two bands index different aspects of task processing. Alpha activity is often linked to attentional gating and the suppression of task-irrelevant processing, such that elevated alpha can be consistent with reduced recruitment of task-relevant sensorimotor circuitry under high demand (Pfurtscheller and Lopes da Silva, 1999; Jensen and Ali, 2010). The alpha increase observed under VF sinusoid is consistent with cognitive load theory: when visual-only guidance was paired with the higher complexity trajectory, the combined attentional demands may have exceeded available processing resources, reducing effective engagement of task-relevant sensorimotor circuits, a pattern reflected in poorer movement outcomes and elevated physiological load under this condition. Beta desynchronization, by contrast, is commonly associated with task-related sensorimotor processing and updating during movement (Kilavik et al., 2013); the difficulty-driven beta reduction therefore supports the view that higher challenge more reliably recruited these processes, and that VHF may have helped maintain such recruitment under difficulty. Together, the alpha–beta dissociation suggests that feedback modality and task difficulty engage partly distinct neural mechanisms: modality shapes the attentional and cognitive processing of error information (reflected in alpha dynamics), whereas difficulty more directly recruits active sensorimotor updating (reflected in beta dynamics). These findings extend prior work using the same brace-based VR platform, in which VHF was associated with significant post-training increases in both alpha- and beta-band power, together with higher motivation and perceived utility ratings (Nataraj et al., 2026). In the present study, EEG responses were further examined across different trajectory-difficulty conditions, revealing that alpha- and beta-band changes varied with the specific combination of ASF modality and task demand. Considered together, these findings suggest that multimodal ASF can modulate cortical activity, whereas the direction and magnitude of the observed EEG changes may depend on the structure and difficulty of the training task. This distinction may inform how VR training protocols can be designed to selectively target cortical engagement without necessarily increasing peripheral physiological burden.

After the individual physiological measures were considered separately, the physiological index provided a complementary summary of the overall magnitude of physiological perturbation across EEG, heart rate, and EMG. This composite measure was not interpreted as a standalone indicator of beneficial or unfavorable response, because it aggregates heterogeneous signals using absolute percentage changes. Instead, the higher physiological index values in the sinusoidal conditions suggest that trajectory complexity elicited broader physiological modulation, complementing the beta-band difficulty effect and supporting the interpretation that the sinusoidal trajectory imposed greater physiological task demand. Beyond this difficulty-related physiological modulation, convergent evidence from movement performance, cardiovascular response, EMG-based control effort, and virtual work pointed toward enhanced post-training motor control efficiency under multimodal feedback. This convergent profile, characterized by greater reductions in normalized path length and jerk, lower heart rate, lower EMG amplitude, and lower virtual work, suggests that multimodal feedback improved task performance while reducing peripheral physiological load and effort-related task cost. In this interpretation, lower EMG amplitude is not treated as a standalone burden marker; rather, because EMG was also the control signal, it indicates that participants required less myoelectric command amplitude to accomplish the same testing task after training. Thus, performance gains were not achieved by increasing command effort or task cost, but were instead accompanied by more economical sensorimotor control. Figure 10 and Table 1 provide an exploratory description of participant-specific best-case profiles. Because normalized path length was used both to identify and evaluate the best-case condition, the path-length comparison is circular by construction, and differences in outcomes that covary with path length may also be inflated. These results therefore cannot establish that participant-specific selection of ASF modality and task difficulty would produce greater cortical engagement or lower peripheral physiological load. Nevertheless, best-case conditions were observed across all four training combinations, although their distribution was uneven. This pattern is consistent with the possibility that no single training configuration is universally optimal and motivates prospective studies in which the best-case condition is selected from one subset of testing trials and evaluated on an independent subset. The multidimensional assessment framework used here provides a candidate set of measures for such future evaluation. The multidimensional assessment framework employed in this study, spanning kinematics, EEG, EMG, cardiovascular activity, and perceptual ratings, provides a candidate set of biomarkers for guiding such individualized adaptation (Guadagnoli and Lee, 2004; Winstein et al., 1994; Salmoni et al., 1984).

The correlation results (Figure 11) provide additional support for the interpretation of the behavioral and EMG-based control-effort findings. The positive associations between post-training path length change and both completion time change and EMG amplitude change, supported by both the linear fits and complementary Spearman correlations, reinforce the internal consistency of the behavioral and neuromuscular findings: conditions that yielded longer, less direct movement paths were accompanied by proportionally greater time-on-task and higher neuromuscular command effort. In the myoelectric-control paradigm, this coupling is mechanistically plausible: more deviated trajectories require more frequent and larger corrective commands, directly increasing muscular activation demand. Crucially, this means that the reductions in path length observed under multimodal feedback were not achieved at the cost of increased effort, but rather reflected genuinely more efficient sensorimotor execution.

Perceptual survey results provide an additional perspective. Optimization of training prescriptions should not target a single dimension (i.e., better performance); instead, it should balance quantifiable performance improvements, physiological responses and participant-reported experience to promote robust and sustainable benefits (Sigrist et al., 2013; Seitz and Dinse, 2007). Perceptual ratings were broadly consistent with the objective findings. Multimodal conditions (VHF) were rated more favorably than visual-only conditions across motivation and perceived utility, with VHF sinusoid receiving the highest endorsement, aligning with the objective finding that this condition also produced the most favorable physiological profile and the greatest jerk reduction. Agency ratings were comparably high across conditions, suggesting that the myoelectric control interface maintained a consistent sense of voluntary control regardless of feedback modality or task difficulty. Notably, the condition associated with the best objective performance–physiology tradeoff (VHF) was also the most positively perceived, suggesting a degree of convergence between subjective experience and objective benefit. Nevertheless, the relatively high and modestly varying perceptual scores across conditions may also indicate that the ASF–difficulty manipulations were not large enough to produce strong subjective separation, or that the single-item ratings were less sensitive to condition differences than the behavioral and physiological measures. Thus, these perceptual ratings should be interpreted as complementary indicators of participant experience rather than as standalone evidence of condition-specific training effects.

Taken together, these findings show that ASF modality and task difficulty influence different dimensions of VR-based motor training response. A multidimensional assessment approach, combining kinematics, EEG, EMG, heart rate, virtual work, and perceptual ratings, may therefore provide a more informative basis for adaptive rehabilitation design than performance metrics alone.

Several limitations should be considered. This study enrolled a modest, sex-imbalanced neurotypical cohort (N = 14; 10 females, 4 males), limiting statistical power and generalizability to clinical populations. The findings also reflect short-term pre-to-post changes from a single session, without longitudinal follow-up to determine retention. Although training-trial count was matched across conditions, exposure was not normalized by prescribed path length, movement duration, or cumulative movement demand; therefore, difficulty-related physiological and EEG effects should be interpreted as responses to overall trajectory-based task demand rather than cognitive difficulty alone. No formal washout block was included, so residual carryover, condition-order, or within-session learning effects cannot be fully excluded despite randomization, rest breaks, and condition-specific baselines. The descriptive sequence distribution further showed that participant-specific best-case conditions occurred more frequently in the third and fourth positions than in the first two positions. This pattern may be consistent with within-session familiarization, learning, or residual carryover; however, it cannot be separated from condition-specific responses or random variation in the present modest sample.

The participant-specific best-case analysis was exploratory. Because the best-case condition was identified from the same data used to evaluate it, these comparisons cannot distinguish genuine participant-specific responses from bias introduced by selecting the most favorable of four conditions. A conclusive test would identify each participant’s best-case condition from one subset of testing trials and evaluate the associated outcomes on an independent, held-out subset. The number of testing trials in the present protocol was insufficient to support such a split. Future studies should allocate additional testing trials to permit cross-validated selection, which would also allow the stability of each participant’s optimal ASF–difficulty pairing to be assessed directly.

Future work should test larger, sex-balanced cohorts, including participants with neurological impairments, across multi-session protocols. Analytical refinements should include normalized or weighted physiological indices; advanced EEG artifact correction and channel-specific, lateralized, or coherence-based analyses; and MVC-normalized or muscle-specific EMG features. Larger studies should also examine subjective–objective associations among perceptual, behavioral, and physiological task-cost outcomes. Finally, platform extensions could incorporate explicit visuomotor perturbations, such as reversal, translation, or rotation paradigms, together with objective or implicit sense-of-agency measures and portable inertial sensors for adaptive VR-training applications.

## Data Availability

The raw data supporting the conclusions of this article will be made available by the authors, without undue reservation.
